# Biological evaluation, docking studies, and *in silico* ADME prediction of some pyrimidine and pyridine derivatives as potential EGFR^WT^ and EGFR^T790M^ inhibitors

**DOI:** 10.1080/14756366.2022.2135512

**Published:** 2022-11-01

**Authors:** Tarfah Al-Warhi, Ahmed A. Al-Karmalawy, Ayman Abo Elmaaty, Maha A. Alshubramy, Marwa Abdel-Motaal, Taghreed A. Majrashi, Medhat Asem, Ahmed Nabil, Wagdy M. Eldehna, Marwa Sharaky

**Affiliations:** a Department of Chemistry, College of Science, Princess Nourah bint Abdulrahman University, Riyadh, Saudi Arabia; b Pharmaceutical Chemistry Department, Faculty of Pharmacy, Ahram Canadian University, Giza, Egypt; c Department of Medicinal Chemistry, Faculty of Pharmacy, Port Said University, Port Said, Egypt; d Department of Chemistry, College of Science, Qassim University, Buraydah, Saudi Arabia; e Chemistry Department, Faculty of Science, Mansoura University, Mansoura, Egypt; f Department of Pharmacognosy, College of Pharmacy, King Khalid University, Abha, Saudi Arabia; g College of Engineering and Information Technology, Onaizah Colleges, Al-Qassim, Saudi Arabia; h Research Center for Functional Materials, National Institute for Materials Science (NIMS), Tsukuba, Japan; i Biotechnology and Life Sciences Department, Faculty of Postgraduate Studies for Advanced Sciences (PSAS), Beni-Suef University, Beni-Suef, Egypt; j Department of Pharmaceutical Chemistry, Faculty of Pharmacy, Kafrelsheikh University, Kafrelsheikh, Egypt; k School of Biotechnology, Badr University in Cairo, Badr City, Egypt; l Cancer Biology Department, Pharmacology Unit, National Cancer Institute (NCI), Cairo University, Cairo, Egypt

**Keywords:** Pyridine/pyrimidine derivatives, EGFR, *in vitro*, *in silico*

## Abstract

Herein, a set of pyridine and pyrimidine derivatives were assessed for their impact on the cell cycle and apoptosis. Human breast cancer (MCF7), hepatocellular carcinoma (HEPG2), larynx cancer (HEP2), lung cancer (H460), colon cancers (HCT116 and Caco2), and hypopharyngeal cancer (FADU), and normal Vero cell lines were used. Compounds **8** and **14** displayed outstanding effects on the investigated cell lines and were further tested for their antioxidant activity in MCF7, H460, FADU, HEP2, HEPG2, HCT116, Caco2, and Vero cells by measuring superoxide dismutase (SOD), malondialdehyde content (MDA), reduced glutathione (GSH), and nitric oxide (NO) content. Besides, Annexin V-FITC apoptosis detection and cell cycle DNA index using the HEPG-2 cell line were established on both compounds as well. Furthermore, compounds **8** and **14** were assessed for their EGFR kinase (Wild and T790M) inhibitory activities, revealing eligible potential. Additionally, molecular docking, ADME, and SAR studies were carried out for the investigated candidates.

## Graphical Abstract

**Figure:**
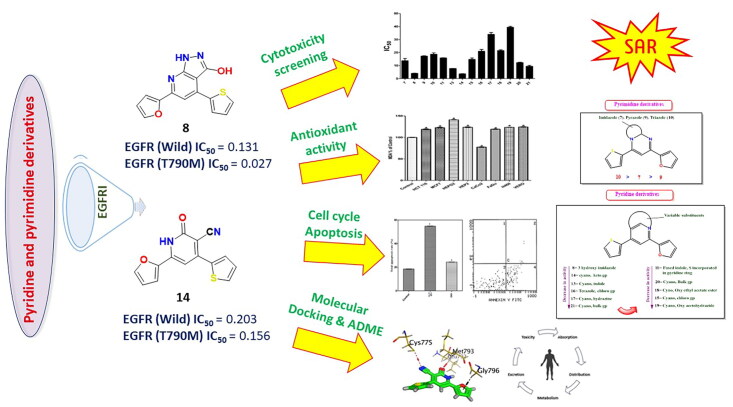

## Introduction

Being the second leading cause of mortality globally, cancer kills roughly 8 million people each year. Additionally, cancer incidence is expected to elevate regrettably by more than 50% in upcoming years^1–3^. Besides, different cancer types have developed acquired chemotherapeutic resistance over the last few decades^4–6^. Furthermore, chemotherapeutics utilised could induce cytotoxicity to other healthy normal cells owing to their poor selectivity. Thus, severe adverse effects may be experienced, such as anaemia, nausea, alopecia, and immunosuppression^7^^,^^8^. As a result, researchers should dedicate their efforts to ice breaking and discovering more appropriate chemotherapeutics, mainly for the most invasive tumours^9^^,^^10^.

Furthermore, cellular functions such as metabolism, survival, apoptosis, and cell proliferation could be regulated by protein kinases (PKs)^11^^,^^12^. Many diseases, including cancer, are caused by disrupting cell signalling cascades *via* kinase alterations, particularly hyper-activation, or mutations^13^^,^^14^.

Epidermal growth factor receptor (EGFR) is regarded as one of the most outstanding PKs, which play a critical function in cell migration and proliferation^15^^,^^16^. Molecules that may affect the control of cancer cell proliferation are targeted by modern-designed molecular strategies. These strategies are capable of improving cancer therapy efficiency more than conventional chemotherapy. Therefore, EGFRs are regarded as outstanding targets for the design of new anti-tumour agents^17^^,^^18^.

An important factor connecting environmental toxicity to the multistage carcinogenic process is oxidative stress. Responses to both endogenous and external stimuli result in the formation of reactive oxygen species (ROS). An intrinsic antioxidant defence system exists to regulate ROS-mediated harm. However, oxidative stress emerges when oxidation surpasses the regulatory systems. Numerous macromolecular components, including DNA, lipids, and proteins, undergo harmful changes as a result of chronic and cumulative oxidative stress. The increased cellular ROS levels are mediated through an alternative strategy through antioxidant use for the sake of tumour cell depletion from ROS-induced survival signalling pathways. It was revealed that increased intracellular ROS levels may be involved in early events of cancer initiation and progression. These treatments might also have a preventative purpose^19^.

In comparison to normal healthy cells, cancer cells exhibit a higher rate of ROS generation and a different redox environment. The majority of chemotherapeutic drugs increase intracellular ROS levels and can change cancer cells’ redox balance^20^.

**Figure 1.**
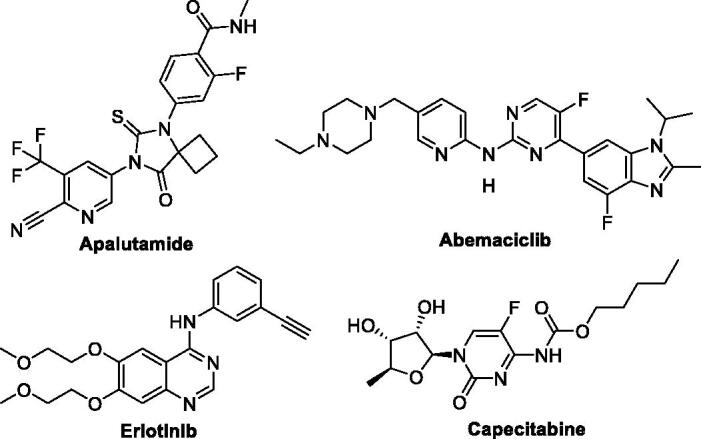
Pyridine and pyrimidines-tethered antitumor drugs.

On the other hand, pyrimidine and pyridine-related compounds are an important class of heterocycles owing to their wide chemical and biological applications. They have been employed widely in the areas of medicine, and material science^21–23^. They are responsible for various biological significance, such as anti-inflammatory^24^, antipyretic^25^, antihypotensive^26^, anticonvulsant^27^, antiviral^28^, antimicrobial^29^, and antidiabetic activities^30^. Among this relevance also, a literature survey revealed that a variety of fused pyrimidines have been reported to be extremely potent anticancer activity against various cell lines^31–33^. Some 2-pyridone derivatives acting as PK inhibitors could exhibit potent anticancer activity^34^. Besides, cyanopyridines revealed potent PIM‐1 inhibitions and PDE3A inhibition^35^^,^^36^. Hamajima et al. reported pyrazolopyridine as having high PI3Kd inhibitory activity with eligible selectivity and oral availability in mice^37^. Additionally, the cell lines A431a, HCT116, and SNU638b were employed for assessing the *in vitro* antiproliferative activity of pyrido[2,3-d]pyrimidine derivatives in addition to its inhibition potential for CDK4/Cyclin D, CDK2-Cyclin A, and EGFR enzyme^38^. Orlikova et al. reported pyrazolopyridine derivatives to reflect their selective cytotoxic potential against K562 cancer cells upon comparison to normal cells^39^. Given their significant cytotoxicity against various cell lines, therefore, pyridines, and pyrimidines were incorporated into many FDA-approved anticancer drugs (Figure 1).

Because of the previous findings and our ongoing research regarding the synthesis of pharmaceutically important pyridines and pyrimidines^40^, the impact of the most active compounds was investigated on the cell cycle and apoptosis by the tumour suppressor p53 to put eyes on their effects on cancer biology assuring the proposed mechanism of action. The current work sheds light on the utility of studied compounds as lead compounds for further investigations as anticancer agents.

**Figure 2.**
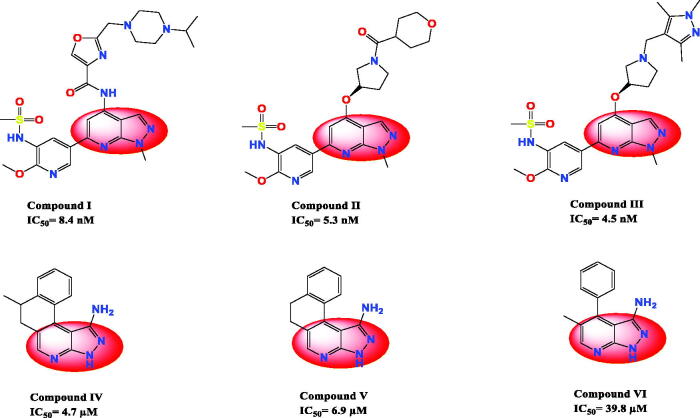
Some reported pyrazolopyridine derivatives and their IC_50_ values as anticancer and kinase inhibitors.

PKs are one of the most important families contributing to a large number of diseases like inflammation, diabetes, and/or cancer^41^. PKs constitute one of the apparent and attractive targets for the treatment of many diseases as they regulate a lot of cellular functions, such as apoptosis, proliferation, metabolism, survival, cell cycle, and DNA damage/repair^42^. The literature revealed some promising pyrazolopyridine derivatives (compounds I–III) reported by Hamajima et al. with inhibitory potential against PI3Kd^37^. Besides, some promising pyrazolopyridine derivatives (compounds IV–VI) were reported by Orlikova et al. with inhibitory potential against NF-_k_B^39^ (Figure 2).

**Figure 3.**
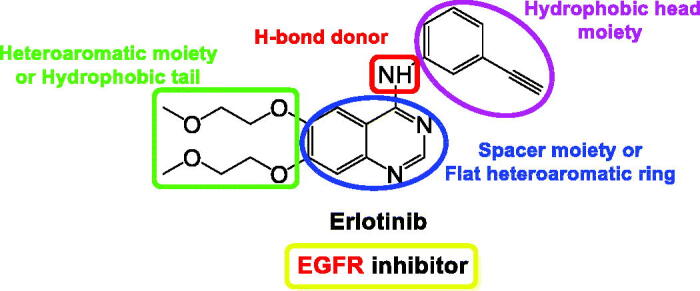
The common pharmacophoric properties of the FDA-approved EGFRI (erlotinib).

Moreover, EGFR is one of the outstanding tyrosine kinase receptors. It regulates several pathways of signal transduction to regulate cell proliferation and apoptosis. Also, it is overexpressed in many cancer types, such as ovarian, colon, and breast by activating the process of angiogenesis^43^. EGFR inhibitors (EGFRIs), such as erlotinib (Figure 3) were approved by the FDA in 2004 for clinical use as an anticancer drug^44^.

The common pharmacophoric properties of EGFRI (erlotinib) are depicted in Figure 3. The first one is the presence of a hydrophobic moiety to act as a head occupying the first hydrophobic region. The second feature is the presence of an H-bond donor in the spacer region occupying the linker region between the adenine binding region and the hydrophobic region I. The third pharmacophoric feature of EGFRIs is required to be a flat heteroaromatic moiety to be able to occupy the binding pocket of adenine (hinge segment). Moreover, a second heteroaromatic or hydrophobic moiety is required to act as a tail, occupying EGFR’s second hydrophobic region^45–48^.

### Rationale-based design

Relying on the basic pharmacophoric properties of EGFRIs represented in Figure 3, we decided to propose the tested pyrimidine and pyridine derivatives as potential EGFRIs.

Guided by the above-discussed pharmacophoric features of EGFRIs which are a hydrophobic moiety to act as a head occupying the first hydrophobic region, an H-bond donor in the spacer region occupying the linker region between the adenine binding region and the hydrophobic region I, a flat heteroaromatic moiety to be able to occupy the binding pocket of adenine (hinge segment), and a second heteroaromatic or hydrophobic moiety to act as a tail and staying at EGFR’s second hydrophobic region^45^.

**Figure 4.**
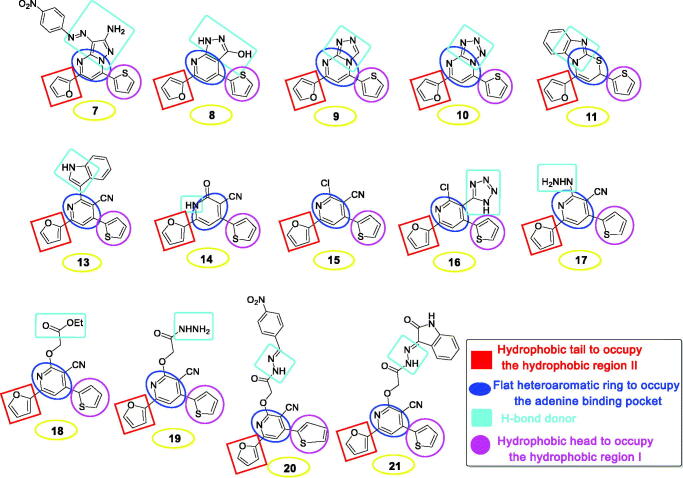
Schematic illustration disclosing the pharmacophoric features of the afforded pyrimidine and pyridine derivatives as EGFRIs.

Herein, the rationale-based design was based on the presence of a pyrimidine or pyridine ring to be inserted into the binding pocket of adenine and act as a flat heteroaromatic moiety. Also, both the thiophene and furan rings were proposed to act as a head to occupy the first hydrophobic region and a tail to occupy the second hydrophobic region of EGFR, respectively. However, the second pharmacophoric feature, which is an H-bond donor in the spacer region, was observed to be either amino, hydroxy, carboxy, or protonated nitrogen atom (Figure 4).

## Results and discussion

### Chemistry

**Scheme 1.**
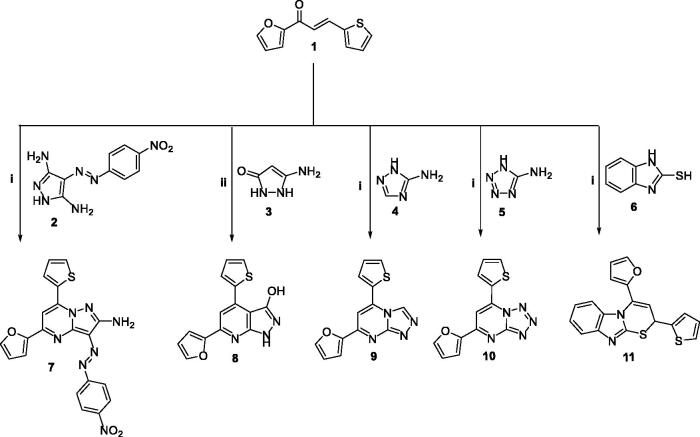
The detailed synthesis of the afforded fused pyrimidine derivatives (**7–11**); Reagents and conditions: i) KOH, DMF, reflux; ii) Pyridine, reflux.

**Scheme 2.**
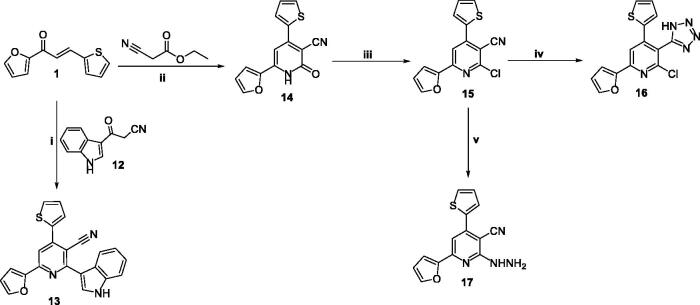
The detailed synthesis of the pyridine derivatives (**14–17**); Reagents and conditions: i) Amm. acetate, Acetic acid, reflux 12 h; ii) Amm. acetate, butanol, reflux 5 h; iii) POCl_3_, PCl_5_, heating 10 h; iv) NaN_3_, DMF, reflux 8 h; ii) NH_2_NH_2_, dioxane, reflux 12 h.

**Scheme 3.**
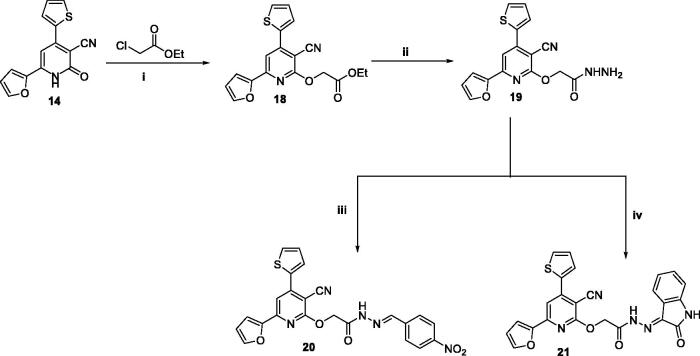
The detailed synthesis of the afforded pyridine candidates (**18–21**); Reagents and conditions: i) DMF, K_2_CO_3_, reflux 8 h; ii) Ethanol, reflux 6 h; iii) Ethanol, Acetic acid, reflux 2 h (ii) Ethanol, reflux 2 h.

The pyridine and pyrimidine derivatives (**7–21**) have been prepared by the general synthetic routes (Schemes 1–3). The chalcone1-(furan-2-*yl*)-3-(thiophen-2-*yl*) chalcone **1**^40^ was synthesised and utilised as an intermediate to get the desired targets. Initially, compounds **(7–17)** were prepared through the treatment of **1** with various primary heteroaryl amines attached at the α-site related to the ring nitrogen (1,3-*N,N* nucleophiles) **(2–5)**, namely: 4-((4-nitrophenyl)diazenyl)-*1H*-pyrazole-3,5-diamine, 5-amino-1, 2-dihydro-3*H*-pyrazolo-3-one, 3-amino-1,2,4-triazole, and 5-amino-1,2,3,4-tetrazole monohydride, respectively, in refluxing DMF and in presence of Alc. KOH (Scheme 1). Otherwise, **8** was also prepared in pyridine. Also, compound **1** was cyclised with 2-mercaptobinzemidzole **6** in the same basic conditions. Pyridine derivatives (**13** and **14**) were synthesised in good yield by further cyclisation of chalcone **4** with 3-cyanoacetyl indole, ethylcyanoacetate, or cyanoacetamide, respectively. Consequently, the nucleophilic substitution of the 2-pyridone derivative **14** with chlorine using POCl_3_/PCl_5_ to afford the 2-chloropyridine derivative **15** which by subsequent cyclisation with sodium azide furnished the tetrazolo derivative **16** in a 53% yield. While condensation of **15** with hydrazine hydrate in dioxane yielded the hydrazine derivative **17**.

Finally, the alkylation of **14** with ethyl chloroacetate was carried out under alkaline conditions at room temperature, giving the ethyl acetate derivative **18.** However, compound **18** was used as an intermediate for the synthesis of Schiff bases **20** and **21** firstly *via* its condensation with hydrazine hydrate to give the hydrazide derivative **19** and then treatment of **19** with 4-nitrobenzaldehyde and isatin in boiling ethyl alcohol, respectively, as outlined in Scheme 2. Spectral and analytical measurements were used to confirm their structures.

### Biological evaluations

#### Cytotoxicity screening against human cancer cell lines

It was revealed that all investigated cell lines were affected by the afforded pyrimidine and pyridine derivatives at different concentrations (5, 12.5, 25, and 50 μg/mL). Table 1 and Figure 5 show that samples have IC_50_ on all tested cell lines, which are (MCF7, HEPG2, HEP2, HCT116, Caco2, H460, FaDu, and Vero) after 48 h.

Figure 5.IC_50_ (µg/mL) of all molecules on different human cancer cell lines following 48 h.

**Figure:**
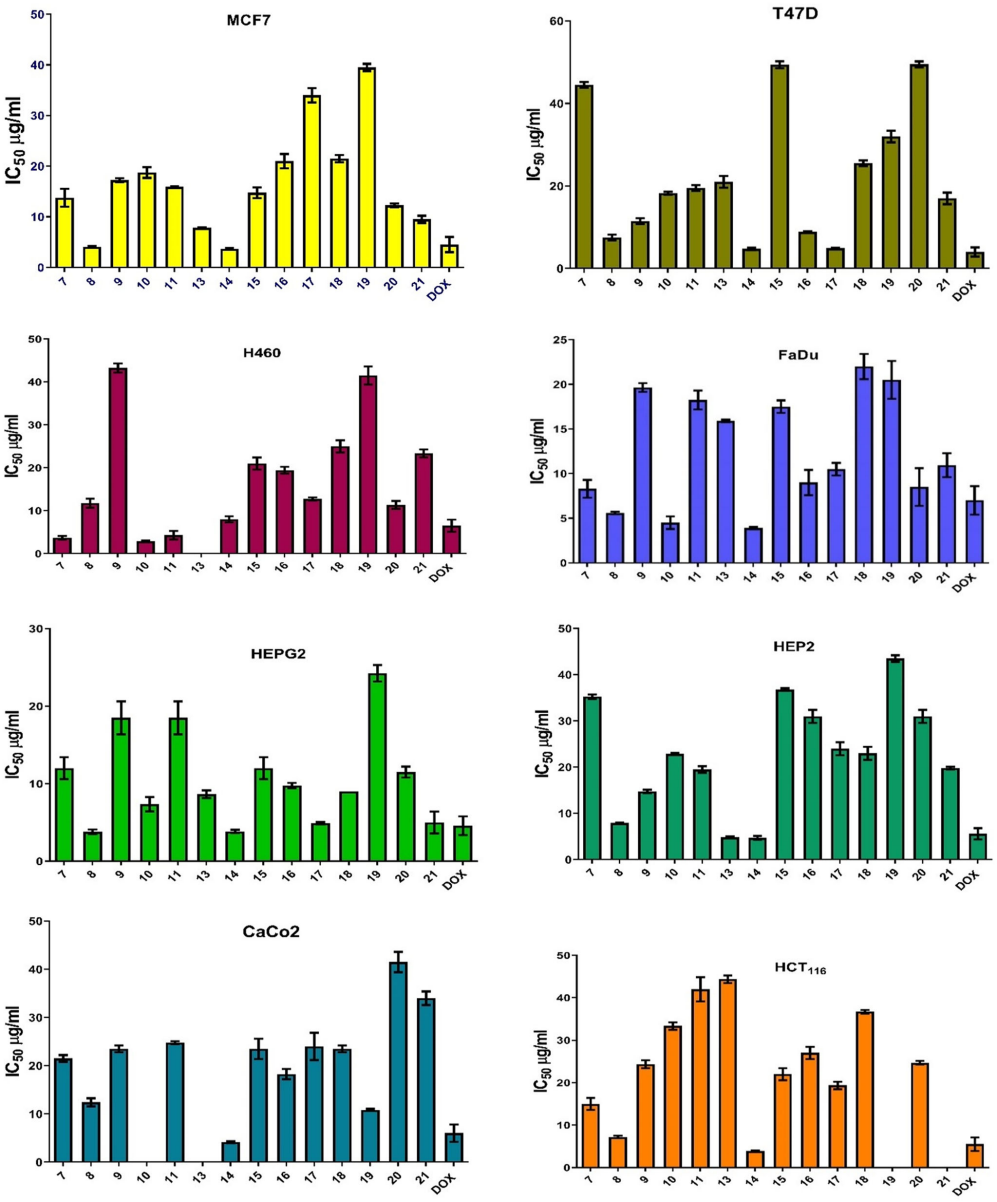

**Figure:**
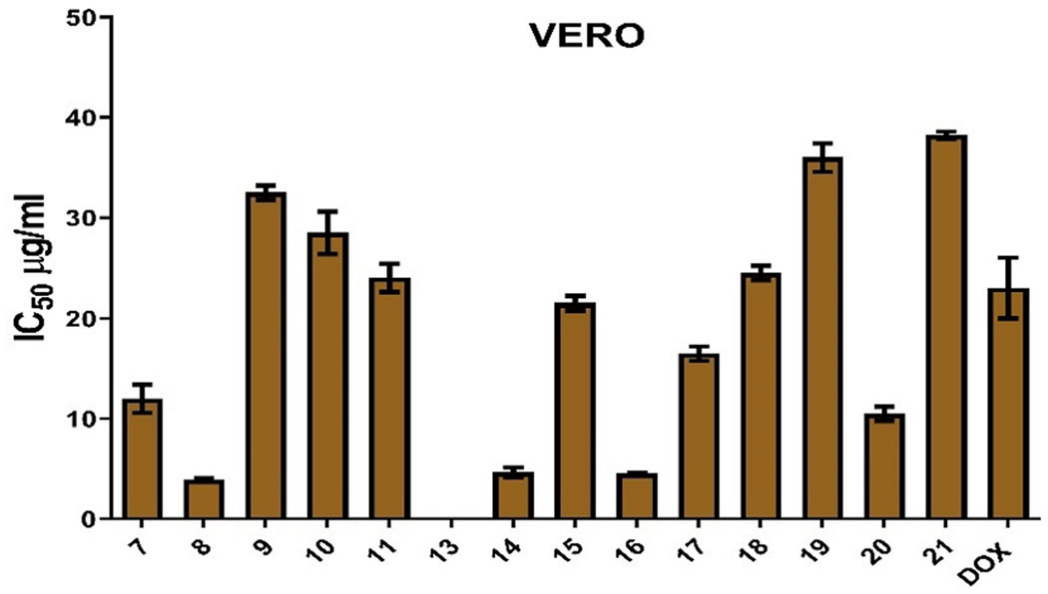

**Table 1.** Pyrimidine and pyridine IC_50_ (µg/mL) on different cell lines.

Sample	MCF7		T47D		VERO		H460		FADU		HEP2		HEPG2		HCT116		CaCo2	
	IC_50_(µg/mL)	SD	IC_50_(µg/mL)	SD	IC_50_(µg/mL)	SD	IC_50_(µg/mL)	SD	IC_50_(µg/mL)	SD	IC_50_(µg/mL)	SD	IC_50_(µg/mL)	SD	IC_50_(µg/mL)	SD	IC_50_(µg/mL)	SD
**7**	13.70	1.76	44.50	0.70	12.00	1.35	3.70	0.42	8.30	0.98	35.20	0.49	12.00	1.41	15.00	1.41	21.50	0.70
**8**	4.10	0.14	7.50	0.70	3.90	0.14	11.70	1.06	5.60	0.14	7.90	0.14	3.80	0.28	7.20	0.28	12.40	0.84
**9**	17.20	0.35	11.50	0.70	32.50	0.70	43.20	1.06	19.60	0.49	14.70	0.35	18.50	2.12	24.30	0.91	23.50	0.70
**10**	18.70	1.06	18.20	0.35	28.50	2.12	2.90	0.14	4.50	0.70	22.80	0.21	7.30	0.91	33.30	0.91	--	--
**11**	15.90	0.14	19.50	0.70	24.00	1.41	4.30	0.98	18.20	1.06	19.50	0.70	18.50	2.12	42.00	2.82	24.80	0.28
**13**	7.80	0.07	21.00	1.41	–	–	–	–	15.90	0.14	4.80	0.21	8.60	0.49	44.30	0.91	--	--
**14**	3.70	0.14	4.70	0.29	4.60	0.40	8.00	0.70	3.90	0.14	4.70	0.42	3.80	0.21	3.90	0.14	4.10	0.21
**15**	14.70	1.06	49.40	0.84	21.50	0.70	21.00	1.41	17.50	0.70	36.80	0.28	12.00	1.45	22.00	1.41	23.50	2.12
**16**	21.00	1.41	8.90	0.14	4.50	0.07	19.40	0.77	9.00	1.41	31.00	1.42	9.70	0.35	27.00	1.41	18.20	1.06
**17**	34.00	1.41	4.90	0.14	16.50	0.70	12.70	0.35	10.50	0.70	24.00	1.41	4.90	0.14	19.30	0.91	24.00	2.82
**18**	21.50	0.70	25.50	0.70	24.50	0.70	25.00	1.41	22.00	1.41	23.0	1.40	9.00	0.03	36.70	0.42	23.50	0.70
**19**	39.50	0.70	32.00	1.41	36.00	1.31	41.50	2.12	20.50	2.12	43.50	0.70	24.20	1.06	–	–	10.80	0.21
**20**	12.20	0.35	49.50	0.70	10.50	0.71	11.30	0.91	8.50	2.12	31.00	1.41	11.50	0.70	24.70	0.42	41.50	2.12
**21**	9.50	0.70	17.00	1.31	38.20	0.35	23.30	0.91	10.90	1.34	19.80	0.28	5.00	1.41	–	–	34.00	1.41
**DOX**	4.50	1.50	4.00	1.10	23.00	3.00	6.50	1.40	7.00	1.60	5.60	1.20	4.58	1.20	5.50	1.60	6.00	1.80

Hence, the IC_50_ concentrations of **8** and **14** in all cell lines were employed in all the following mechanistic experiments. Therefore, MCF7 3.80 and 7.00 µg/mL, HEPG2 4.00 and 3.60 µg/mL, HEP2 4.40 and 8.00 µg/mL, HCT116 4.00 and 7.40 µg/mL, Caco2 4.30 and 11.80 µg/mL, H460 8.50 and 12.50 µg/mL, FaDu 3.80 and 5.70 µg/mL, and Vero 4.30 and 4.00 µg/mL of compounds (**8** and **14**), respectively, were used.

Data are represented as the mean of surviving fraction ± SD of three independent experiments performed in five replicates.

From these findings, both compounds **8** and **14** have been chosen to be tested for their antioxidant activities. On the other hand, the antioxidant activities of **8** and **14** were investigated using the most sensitive cell line.

Data are represented as the mean of surviving fraction ± SD of three independent experiments performed in five replicates.

#### Antioxidant activity

Consequently, both compounds **8** and **14** were tested for their antioxidant activity in MCF7, H460, FADU, HEP2, HEPG2, HCT 116, Caco2, and Vero cells, and the dose used was the IC_50_ in all cell lines.

**Figure 6.**
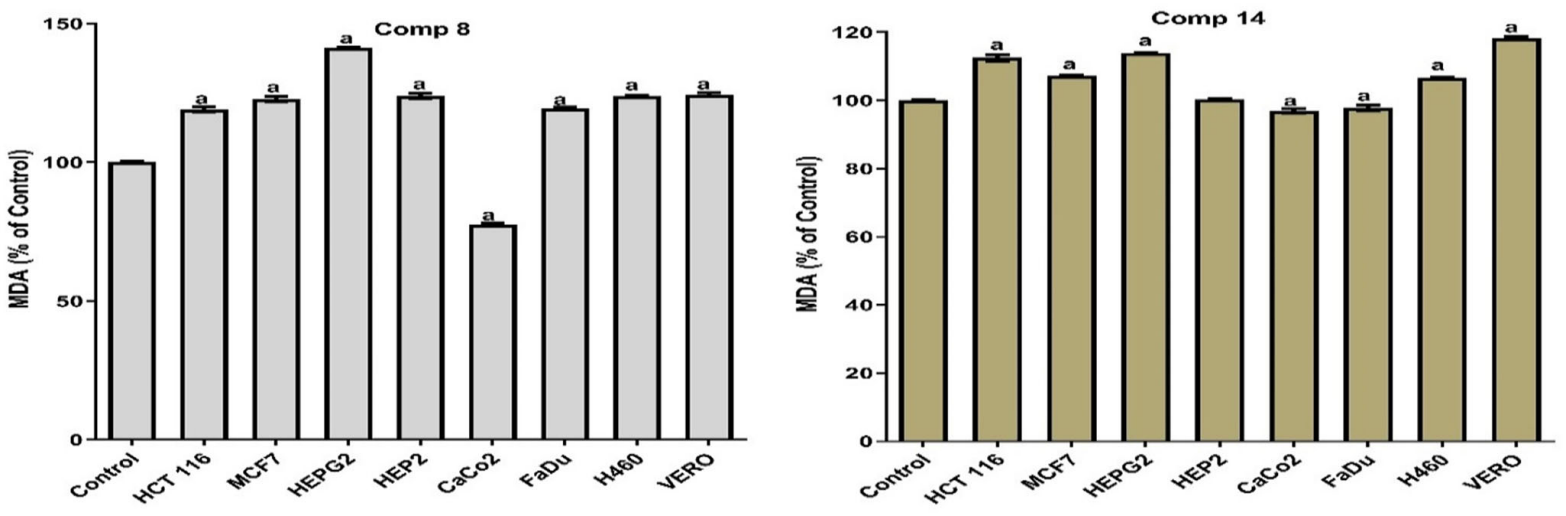
Results of compounds **8** and **14** on MDA in cell lyses of all tested cells following 48 h.

**Figure 7.**
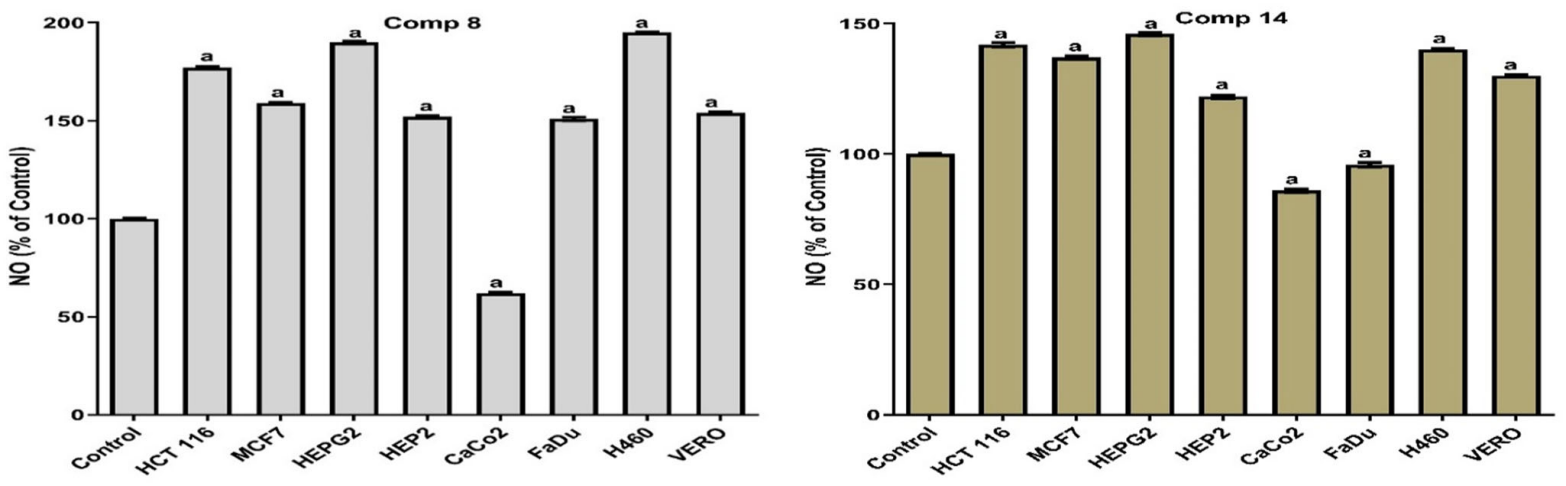
Results of compounds **8** and **14** on NO in culture media of all tested cells following 48 h.

**Figure 8.**
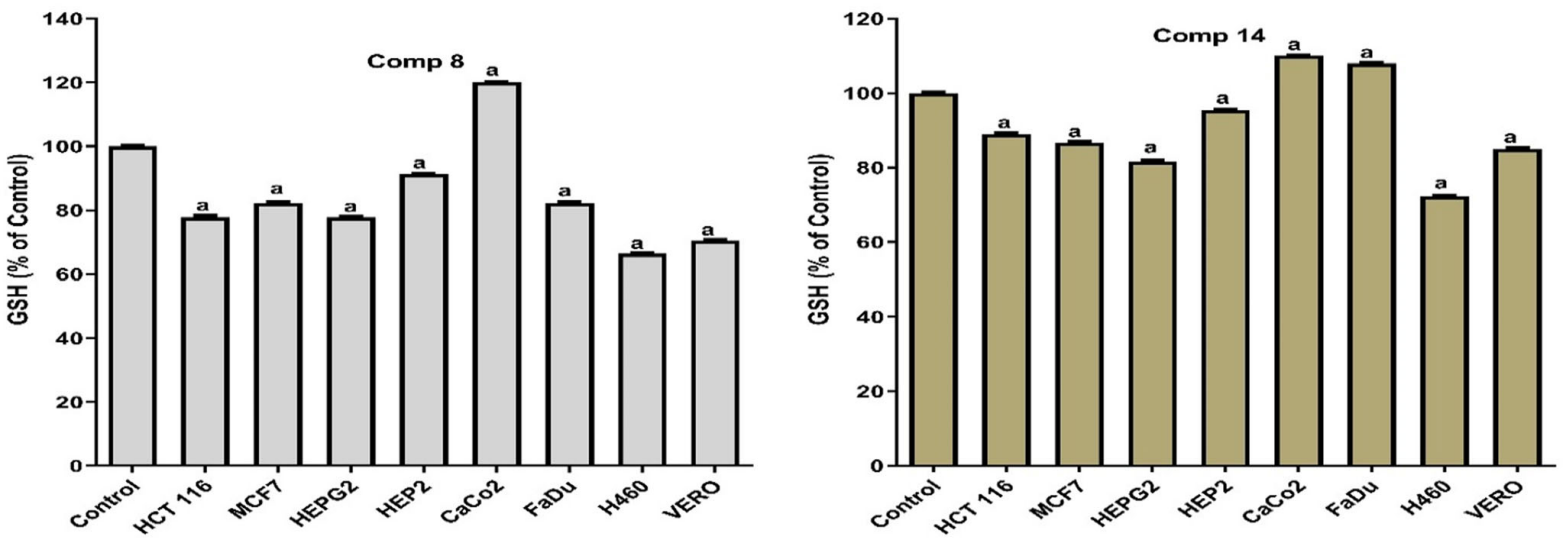
Results of compounds **8** and **14** on GSH in cell lyses of all tested cells following 48 h.

**Figure 9.**
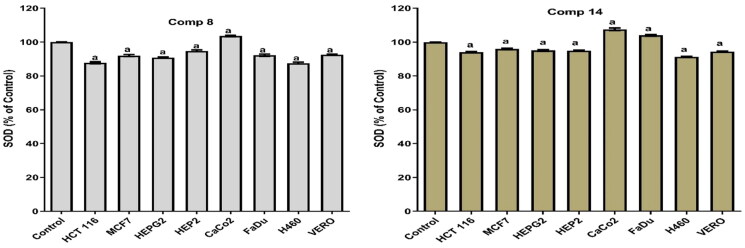
Results of compounds **8** and **14** on SOD in cell lyses of all tested cells following 48 h.

Notably, compound **14** produced a pro-oxidant effect in MCF7, H460, HEP2, HEPG2, HCT, and Vero cells by significantly increasing MDA and NO with an apparent decrease in the levels of SOD and GSH. Moreover, compound **14** achieved an anti-oxidant effect in FADU and Caco2. This was confirmed by the increase in the SOD and GSH levels with the decrease in the levels of MDA and NO. Also, compound **8** showed a pro-oxidant effect in all tested cell lines except Caco2 where it produced an antioxidant effect. It significantly increased the MDA and NO levels with an apparent decrease in the levels of SOD and GSH (pro-oxidant). At the same time, it increased the levels of SOD and GSH and decreased the levels of MDA and NO (antioxidant) in the Caco2 cell line as shown in Figures 6–9.

#### Apoptosis activity

**Figure 10.**
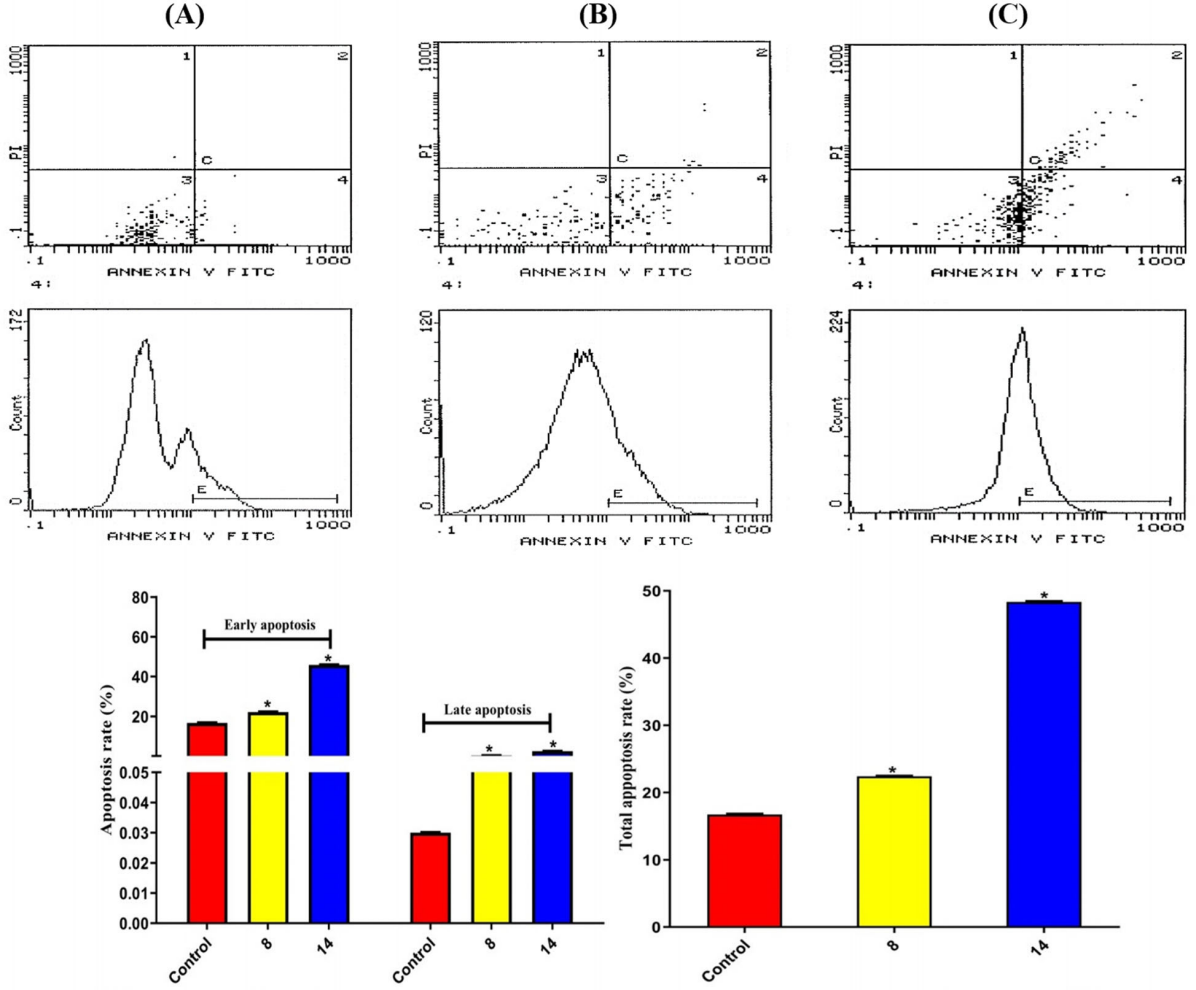
Results of compounds **8** and **14** on apoptosis in HEPG2 cells following 48 h. (A) Control, (B) Compound **8**, and (C) Compound **14**.

Furthermore, both compounds **8** and **14** were evaluated for apoptosis using flow cytometry on the HEPG2 cell line. The % rate of total apoptosis for control, compound **8**, and compound **14** were 16.73, 22.44, and 48.36 (%), respectively. Notably, both **8** and **14** derivatives showed an apparent increase in the total, early, and late % rates of apoptosis with respect to the control (*p* = 0.0001) (Figure 10).

#### Cell cycle analysis

**Figure 11.**
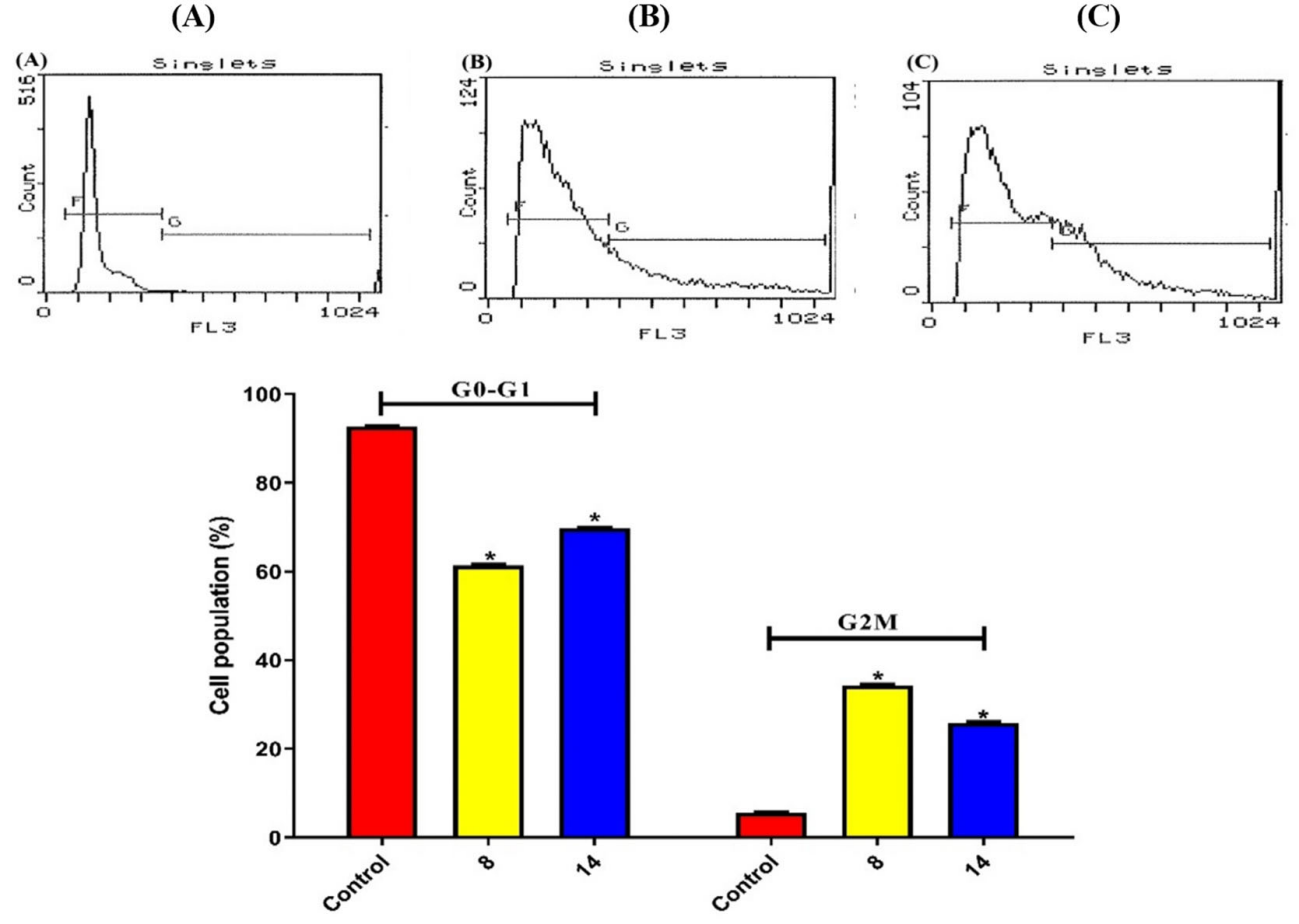
Results of compounds **8** and **14** on the cell cycle of HEPG2 following 48 h. (A) Control, (B) Compound **8**, and (C) Compound **14**.

Furthermore, both **8** and **14** compounds were investigated for their effects on the cell cycle in HEPG2 cells. There was a significant decrease from control (92.7%) in G0/G1 with derivatives **8** (61.4%) and **14** (69.8%) (*p* = 0.0001). Briefly, it was clear that an apparent increase from control (5.57%) in the G2/M phase was observed for both **8** (34.4%) and **14** (25.9%) derivatives (*p* = 0.0001) (Figure 11).

#### EGFR kinase (wild and T790M) inhibition assay

**Figure 12.**
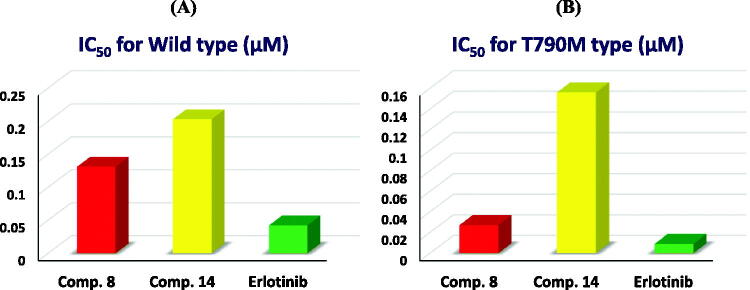
The bar chart representation reveals the inhibitory levels of the assessed derivatives against (A): EFGR Wild and (B): EGFR T790.

EGFR plays a pivotal role in tumorigenesis. Hence, cancer treatment targeting the EGFR gene has shown great progress. However, not all cancer patients are sensitive to EGFR-tyrosine kinase inhibitors and that could be attributed to EGFR gene mutation^49^. So, it is important to reveal the efficacy of our investigated compounds against both non-mutagenic EGFR (EGFR-wild type) and mutagenic EGFR (EGFR-T790M). Consequently, the most active compounds (**8** and **14**) that displayed outstanding anti-proliferative activities towards the utilised cancer cell lines were employed to assess their EGFRI potential. The reagent, Kinase-Glo MAX, was used^50^, and luminescence was detected by applying the microplate reader. Erlotinib was used as a reference standard in this experiment as shown in Table 2. Accordingly, considering EGFR kinase wild, it was revealed that the investigated compounds showed less inhibitory potential than erlotinib with IC_50_ values of 0.131 and 0.203 µM for compounds **8** and **14**, respectively, whereas, erlotinib exhibited an IC_50_ value of 0.042 µM. Hence, it was elicited that compounds **8** and **14** experienced eligible inhibitory potential against EGFR kinase (wild) as depicted in Figure 12(A). However, regarding EGFR kinase T790M, it was disclosed that the assessed compounds displayed less inhibitory potential than erlotinib with IC_50_ values of 0.027 and 0.156 µM for compounds **8** and **14**, respectively, whereas, erlotinib exhibited an IC_50_ value of 0.009 µM. Hence, we can deduce that compounds **8** and **14** could display feasible inhibitory potential against EGFR kinase (T790M) as depicted in Figure 12(B).

**Table 2.** The inhibitory potentials of the investigated **8** and **14** targets towards EGFR (Wild) and EGFR (T790M).

Compound	EGFR IC_50_ (µM)	
	Wild	T790M
**8**	0.131 ± 0.008	0.027 ± 0.002
**14**	0.203 ± 0.012	0.156 ± 0.009
**Erlotinib**	0.042 ± 0.003	0.009 ± 0.001

### In silico studies

#### Docking studies

**Figure 13.**
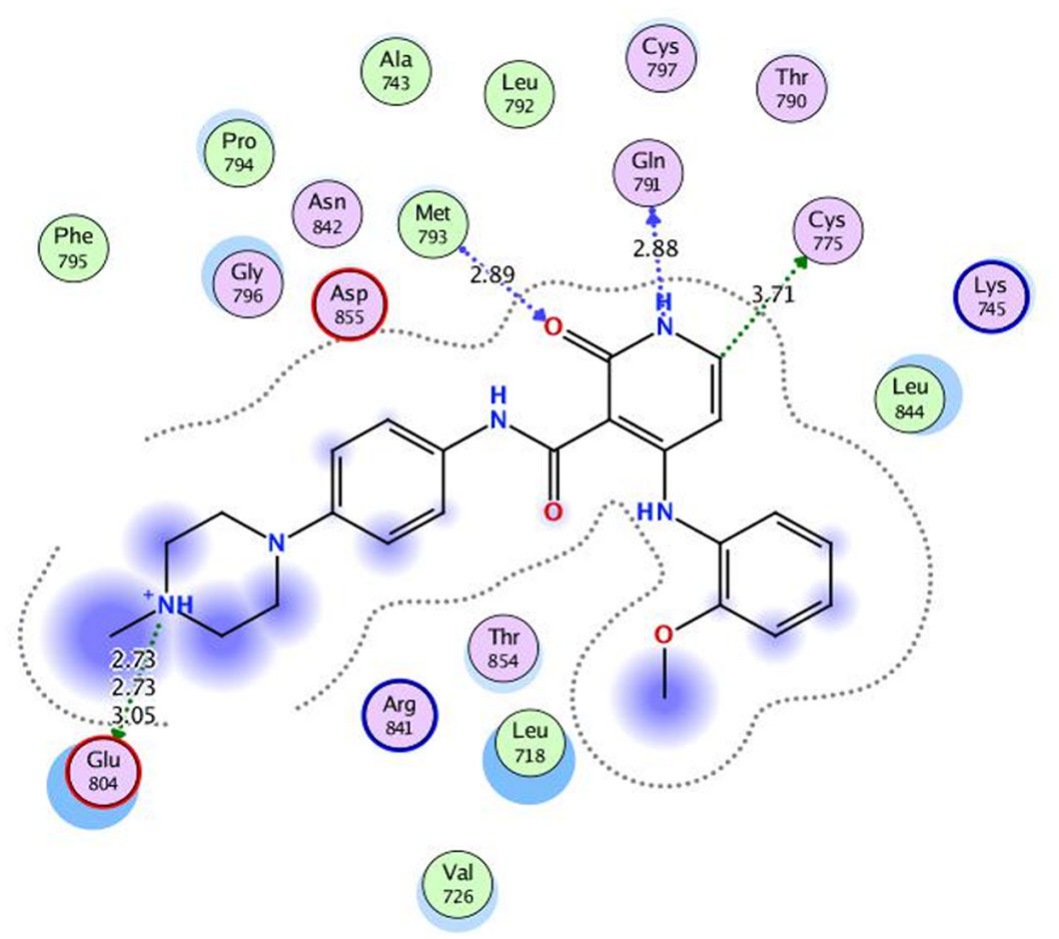
2D binding interactions of co-crystallised **5Q4** undocked ligand at EGFR-kinase domain.

First, the key amino acids required for EGFR-Kinase domain interaction were identified with the aid of co-crystallised downloaded pyridinone ligand (**5Q4**) interactions. It was revealed that **5Q4** forms four hydrogen bonds with Glu-804, Cys-775, Gln-791, and Met-793, and two ionic bonds with Glu-804 at EGFR-Kinase (Figure 13).

Accordingly, it was clear that the tested candidates showed diverse binding scores and modes at the EGFR-Kinase domain receptor compared to that of the co-crystallised **5Q4** inhibitor. Hence, concerning its RMSD values and interactions results, synthesised compounds **8** and **14**, showed favourable results between tested compounds at the EGFR-Kinase receptor. The synthesised compounds **8** and **14** showed binding interactions nearly similar to that attained by the co-crystallised ligand. The chemically synthesised compound **8** revealed binding interaction to EGFR-Kinase domain through forming two hydrogen bonds with Gln-791 and Met-793, and one pi-H bond with Leu-718 with RMSD = 1.6807, whereas, the chemically synthesised compound **14** interactions revealed its binding with Cys-775 and Met-793 through forming H-bonds, and Gly-796 by a pi-H bond with RMSD = 1.1802. However, the docked **5Q4** was capable of forming three hydrogen bonds with Gln-791, Cys-775, and Met-793, and one pi-H bond with Leu-718 with RMSD = 1.3242 (Table 4).

**Table 4.** 3D interactions and positioning of the chemically synthesised compounds (**8** and **14**) and the docked **5Q4** reference at EGFR-Kinase target receptor.

Comp.	3D interactions	3D positioning
**8**	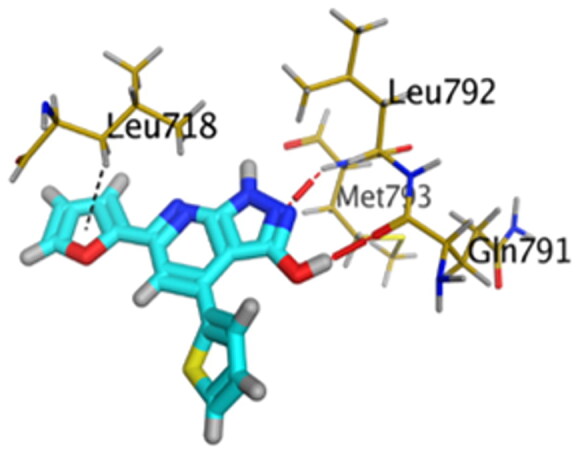	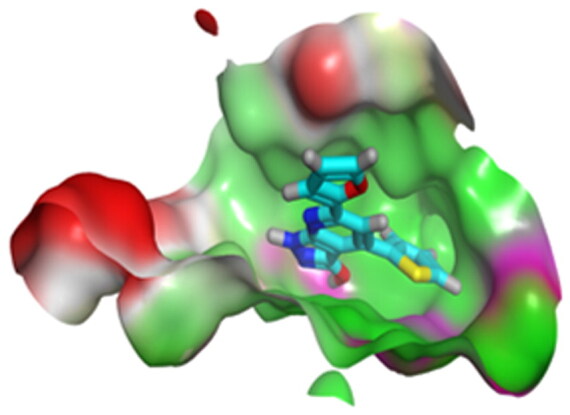
**14**	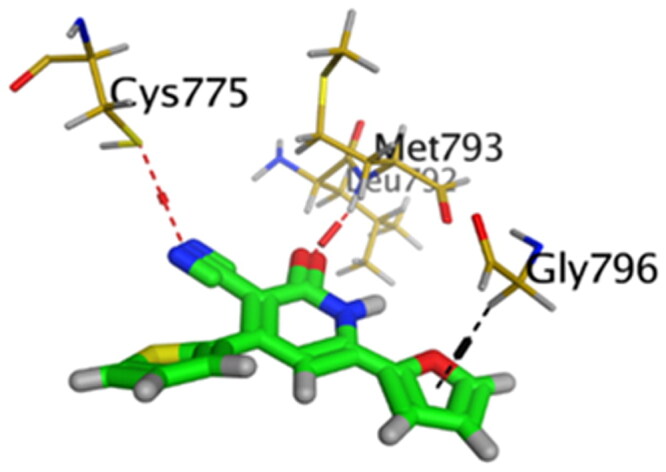	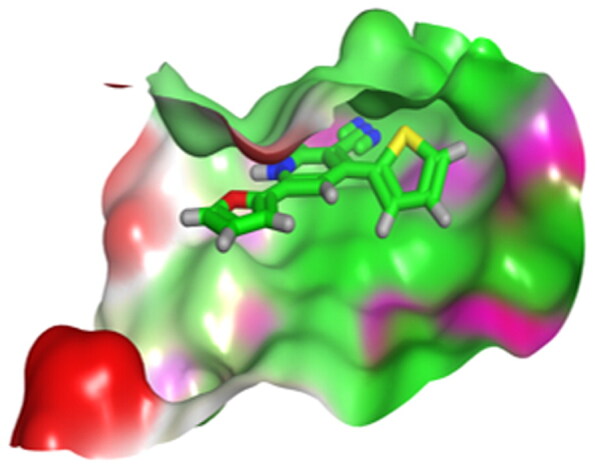
**5Q4**	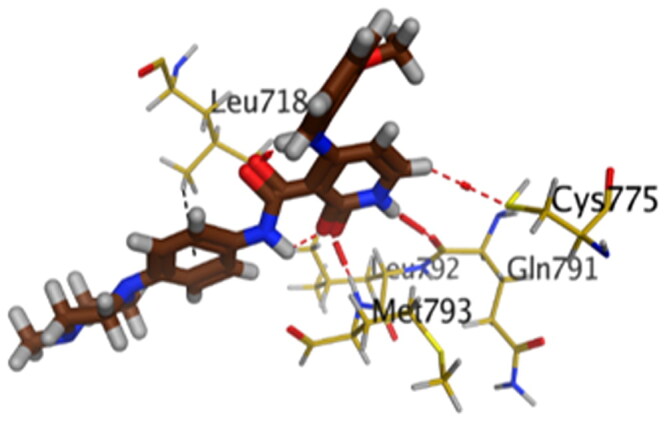	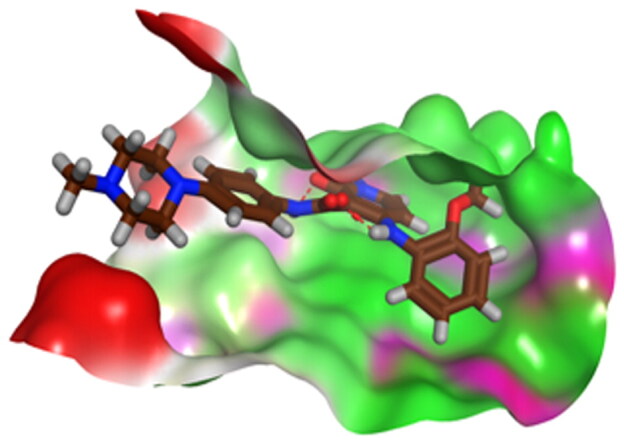

Hydrogen bonds are represented in red and H-pi ones are represented in black.

The docking results of compounds **8**, **14**, and **5Q4** show their interactions and positioning in 3D orientation at the EGFR-kinase domain (Tables 3 and 4). The detailed docking scores, RMSD values, interactions, and visualisation of other candidates are described in the Supplementary data as 2D and 3D interactions, and surface and maps (Tables SI 1–SI 3).

**Table 3.** Receptor-binding energies and interactions of compounds **8,** 1**4**, and **5Q4** into the **5Q4** pocket of EGFR-kinase.

Comp.	S^a^	Amino acid bond	Distance (Å)
**8**	−5.8756	Gln-791 (A) H-donor	2.92
		Met-793 (A) H-acceptor	3.13
		Leu-718 (A) pi-H	4.26
**14**	−5.5083	Cys-775 (A) H-donor	3.52
		Met-793 (A) H-acceptor	3.06
		Gly-796 (A) pi-H	4.02
**5Q4**	−7.0125	Gln-791 (A) H-donor	3.15
		Cys-775 (A) H-donor	4.13
		Met-793 (A) H-acceptor	3.04
		Leu-718 (A) pi-H	3.95

^a^S: Score of a ligand into the binding pocket of **5Q4** (Kcal/mol).

#### In silico physicochemical, pharmacokinetic, and ADME studies

The SwissADME website of the Swiss Institute of Bioinformatics (SIB)^51^ was applied for the estimation and prediction of the physicochemical and pharmacokinetic characteristics of the examined derivatives^52–54^ (Table 5).

**Table 5.** The physicochemical and pharmacokinetic characteristics of the examined derivatives (**7–21**).

Comp.	Log Po/w (WLOGP)	Log S (ESOL)	Water solubility	GI absorption	BBB permeability	P-gp substrate	Metabolic enzymes inhibition	Drug likeness matching
**7**	3.55	−4.93	Moderately soluble	Low	No	No	CYP2C19 inhibitor	Lipinski
**8**	3.65	−3.99	Soluble	High	No	Yes	CYP1A2 inhibitor	Lipinski, Ghose, Veber, Egan, Muegge
							CYP2C19 inhibitor	
**9**	3.11	−3.52	Soluble	High	No	No	CYP1A2 inhibitor	Lipinski, Ghose, Veber, Egan, Muegge
							CYP2C19 inhibitor	
**10**	1.81	−3.32	Soluble	High	No	No	CYP2C19 inhibitor	Lipinski, Ghose, Veber, Egan, Muegge
**11**	5.10	−5.27	Moderately soluble	High	No	Yes	CYP1A2 inhibitor	Lipinski, Ghose, Veber, Egan, Muegge
							CYP2C19 inhibitor	
							CYP2C9 inhibitor	
							CYP3A4 inhibitor	
**13**	6.09	−5.42	Moderately soluble	Low	No	Yes	CYP1A2 inhibitor	Lipinski, Veber, Muegge
							CYP2C19 inhibitor	
							CYP2C9 inhibitor	
							CYP2D6 inhibitor	
							CYP3A4 inhibitor	
**14**	3.24	−3.10	Soluble	High	No	No	CYP1A2 inhibitor	Lipinski, Ghose, Veber, Egan, Muegge
							CYP2C19 inhibitor	
							CYP2C9 inhibitor	
							CYP2D6 inhibitor	
**15**	4.60	−4.40	Moderately soluble	High	No	Yes	CYP1A2 inhibitor	Lipinski, Ghose, Veber, Egan, Muegge
							CYP2C19 inhibitor	
							CYP2C9 inhibitor	
**16**	3.90	−4.42	Moderately soluble	High	No	No	CYP1A2 inhibitor	Lipinski, Ghose, Veber, Egan, Muegge
							CYP2C19 inhibitor	
**17**	3.04	−3.75	Soluble	High	No	Yes	CYP1A2 inhibitor	Lipinski, Ghose, Veber, Egan, Muegge
							CYP2C19 inhibitor	
							CYP2C9 inhibitor	
							CYP2D6 inhibitor	
							CYP3A4 inhibitor	
**18**	3.88	−4.11	Moderately soluble	High	No	No	CYP1A2 inhibitor	Lipinski, Ghose, Veber, Egan, Muegge
							CYP2C19 inhibitor	
							CYP2C9 inhibitor	
							CYP3A4 inhibitor	
**19**	2.31	−3.04	Soluble	Low	No	Yes	CYP1A2 inhibitor	Lipinski, Ghose, Muegge
							CYP2C9 inhibitor	
							CYP3A4 inhibitor	
**20**	4.38	−5.05	Moderately soluble	Low	No	No	CYP2C19 inhibitor	Lipinski, Ghose
							CYP2C9 inhibitor	
**21**	3.22	−5.19	Moderately soluble	Low	No	No	CYP2C19 inhibitor	Lipinski, Ghose
							CYP2C9 inhibitor	

The aim is to confirm that the most favourable compounds in molecular modelling studies (compounds **8** and **14)** are promising candidates regarding their pharmacokinetic properties. Compounds **8** and **14** exhibited predicted wlogP values of 3.65 and 3.24, respectively, with no blood–brain barrier (BBB) permeability and so no CNS side effects are predicted. Both compounds **8** and **14** showed high GIT absorption with reasonable H_2_O solubility. Moreover, compound **8** is a substrate for P-glycoprotein (PGP+) whereas compound **14** is not a substrate for it (PGP−), so it is not subjected to the efflux mechanism as a drug-resistance mechanism used by many tumour cell lines. Also, compound **8** manifests inhibition for the metabolising enzymes (CYP1A2 and CYP2C19) only. Whereas compound **14** is capable of inhibiting CYP1A2, CYP2C19, CYP2C9, and CYP2D6 metabolising enzymes. Compounds **8** and **14** were in agreement with Lipinski, Veber, Ghose, Muegge, and Egan rules.

## Structure–activity relationship (SAR) study

**Figure 14.**
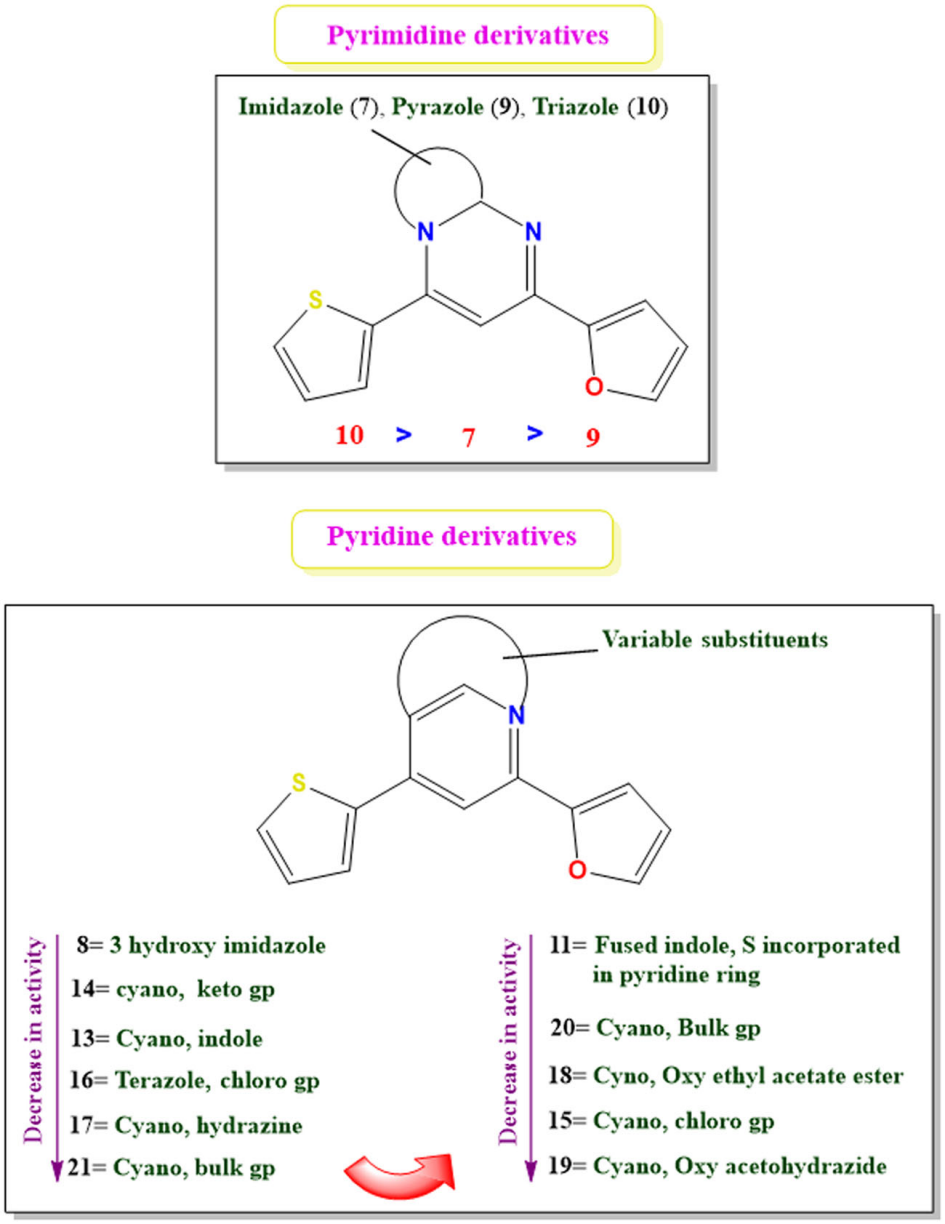
The suggested SAR for the studied pyrimidine and pyridine derivatives.

To attain deep insights and get one step closer to understanding the results of chemical modifications on the activity of studied derivatives on their activities towards the EGFR target site. We decided to conclude and analyse a SAR study based on their effects on the different cell lines used as depicted in Figure 14. The following interesting outcomes were unveiled:

It was revealed that the best activity towards EGFR was attained by pyridine derivatives. In particular, it was found that the best activity was attained by pyridine derivatives with keto and cyano substitutions at positions 2 and 3, respectively (compound **14**), or by fusion with 3-hydroxy pyrazole (compound **8**).

Moreover, eligible activities were attained by some pyridine derivatives retaining imidazole and triazole substituents (compounds **7** and **10**) and pyrimidine derivatives retaining cyano group at position 3 (compounds **13**, **17**, and **21**) or tetrazole ring (compound **16**).

On the other hand, it is worth noting that moderate activities were attained by pyridine derivatives retaining pyrazole (compound **9**) and pyridine derivatives retaining fused indole with sulphur atom incorporated in the pyridine ring (compound **11**), cyano and chloro groups (compound **15**), cyano and oxy ethyl acetate ester (compound **18**), cyano and oxy acetohydrazide (compound **19**), as well as cyano and bulk group (compound **20**).

## Conclusion

Briefly, a series of chemically synthesised pyridine and pyrimidine derivatives were screened for their anticancer activities *via* EGFR inhibition. Among the investigated compounds, both **8** and **14** displayed outstanding effects on the tested cell lines with IC_50_ concentrations of 3.8 and 7 µg/mL (MCF7), 4 and 3.6 µg/mL (HEPG2), 4.4 and 8 µg/mL (HEP2), 4 and 7.4 µg/mL (HCT116), 4.3 and 11.8 µg/mL (CACO), 8.5 and 12.5 µg/mL (H460), 3.8 and 5.7 µg/mL (FaDu), and 4.3 and 4 µg/mL (VERO), respectively. Besides, compound **14** induced a pro-oxidant state in H460, MCF7, HEP2, HEPG2, HCT, and VERO by significantly increasing the MDA and NO. On the other hand, a significant decrease in SOD and GSH levels was observed. Additionally, compounds **8** and **14** achieved a significant increase in apoptosis percentage (total, early, and late) compared to control. Furthermore, there was a significant decrease in the G0/G1 phase with an apparent increase in the S and G2/M phases with both compounds **8** and **14** (*p* = 0.0001). Besides, the EGFR kinase (Wild and T790M) inhibitory potential of the most active compounds (**8** and **14)** assured the rational and the mode of action suggested in this current work. Moreover, the molecular docking study performed ensured the outstanding anticancer activities of the investigated compounds by declaring their binding interactions with the EGFR target receptor. Finally, eligible physicochemical/pharmacokinetics properties, and drug/lead likeness, were obtained.

## Materials and methods

### Chemistry

The reactions’ progress and the compounds’ purity were checked using thin-layer chromatography (TLC), which were monitored using UV light at 365 and 254 nm. Also, all spectral data were recorded according to our previous study^40^.

### Biological evaluations

#### Cytotoxicity screening against human cancer cell lines

In this study, a panel of cell lines was examined for their chemosensitivity. Different concentrations of the synthesised pyridines and pyrimidine derivatives were used in this study (5, 12.5, 25, and 50 µg/mL) for all the examined cell lines. In this study, breast tumour cell line (MCF7), hepatocellular carcinoma cell line (HEPG2), larynx cell line (HEP2), lung cancer cell line (H460), colon cancer cell line (HCT116 and Caco2), hypopharyngeal cell line (FADU), and normal Vero cell line were applied. The full methodology is described in the Supplementary data (SI 1).

#### Oxidative stress assessment

Supplementary data (SI 2).

##### Determination of malondialdehyde content (MDA)

Supplementary data (SI 2(A)).

##### Determination of superoxide dismutase (SOD)

Supplementary data (SI 2(B)).

##### Determination of reduced glutathione (GSH) content

Supplementary data (SI 2(C)).

##### Determination of nitric oxide (NO) content

Supplementary data (SI 2(D)).

#### Cell cycle analysis and apoptosis assay

Liver cancer cells (HEPG2) were seeded in RPMI-1640 media at a density of 250 X 10^3^ cells/mL. Both cell cycle and apoptosis evaluations were performed at 3.8 µg/mL for both compounds **8** and **14**. The detailed method is described in the Supplementary data (SI 3).

#### EGFR kinase (wild and T790M) inhibition assay

The most promising cytotoxic compounds (**8** and **14**) were finally assessed for their inhibitory potential against both EGFR Wild and EGFR T790M. The assay protocol is fully described in the Supplementary data (SI 4).

### In silico studies

#### Docking studies

The MOE 2019.0102^55–57^ was used to examine the binding affinities of the chemically synthesised derivatives on the EGFR through molecular docking. Accordingly, we could reveal the anticancer inhibitory potential of these compounds as promising EGFRIs. Also, the co-crystallised **5Q4** pyridinone inhibitor was inserted in the docking process as a reference standard.

##### Examined compounds preparation

The chemical structures of the examined chemically synthesised compounds were drawn by ChemDraw. Using MOE, the previous structures (**7–21**) were prepared for docking as described earlier^58–60^. The synthesised compounds under investigation and **5Q4** were saved into the same database for the docking step.

##### EGFR-Kinase receptor preparation

The Protein Data Bank was searched to give the crystal structure of the EGFR kinase domain (code: 5EM8)^61^. The preparation process was performed as discussed before^62–64^. The program default items were followed as before^65–67^.

##### Docking of the tested molecules to EGFR-kinase receptor

Docking of the database composed of compounds (**7–17**) and the reference **5Q4** ligand at the EGFR-Kinase domain was carried out. The applied methodology was performed as discussed in detail^68–70^. The MOE specifications were modified as previously mentioned^71–73^. The selected poses were based on their scores and RMSD accordingly^74–76^.

Furthermore, low RMSD values between the conformations of the redocked and crystal **5Q4** ligand indicated a valid performance in a validation process^77–79^.

#### Physicochemical, pharmacokinetic, and ADME studies

The Swiss ADME supplied from the SIB^51^ was used for the physicochemical, pharmacokinetic, and ADME studies of the target compounds^41^,^80^,^81^.

### Statistical analysis

All the previously presented results are the mean ± SD of three separate experiments, which were performed in duplicates. The statistical significance of the results was analysed using one-way ANOVA followed by Tukey’s multiple comparison test. A significantly different from control and *p* < 0.05.

## Supplementary Material

Supplemental Material
