# Supplementary material for: Biological evaluation, docking studies, and in silico ADME prediction of some pyrimidine and pyridine derivatives as potential EGFRWT and EGFRT790M inhibitors
Source: J Enzyme Inhib Med Chem. 2022 Nov 1;38(1):176–91. doi: 10.1080/14756366.2022.2135512 (PMC9635468; doi:10.1080/14756366.2022.2135512)
Supplement: Supplemental Material [file IENZ_A_2135512_SM8497.pdf]

Biological Evaluation, Docking Studies, and in silico ADME Prediction of Some Pyrimidine and Pyridine Derivatives as Potential EGFR^{WT} and EGFR^{T790M} Inhibitors

Tarfah Al-Warhi[a], Ahmed A. Al-Karmalawy[b,*], Ayman Abo Elmaaty[c], Maha A. Alshubramy[d], Marwa Abdel-Motaal[d,e], Taghreed A. Majrashi[f], Medhat Asem[g], Ahmed Nabil[h,i], Wagdy M. Eldehna[j,*], Marwa Sharaky[k]

[a] Department of Chemistry, College of Science, Princess Nourah bint Abdulrahman University, Riyadh, Saudi Arabia.
[b] Pharmaceutical Chemistry Department, Faculty of Pharmacy, Ahram Canadian University, 6th of October City, Giza 12566, Egypt.
[c] Department of Medicinal Chemistry, Faculty of Pharmacy, Port Said University, Port Said 42526, Egypt.
[d] Department of Chemistry, College of Science, Qassim University, Buraydah 51452, Saudi Arabia.
[e] Chemistry Department, Faculty of Science, Mansoura University, Mansoura 35516, Egypt.
[f] Department of Pharmacognosy, College of Pharmacy, King Khalid University, Abha 61441, Saudi Arabia
[g] College of Engineering and Information Technology, Onaizah Colleges, Al-Qassim 56447, KSA.
[h] Research Center for Functional Materials, National Institute for Materials Science (NIMS), 1-1 Namiki, Tsukuba, Ibaraki 305-0044, Japan.
[i] Biotechnology and Life Sciences Department, Faculty of Postgraduate Studies for Advanced Sciences (PSAS), Beni-Suef University, Beni-Suef, Egypt.
[j] Department of Pharmaceutical Chemistry, Faculty of Pharmacy, Kafrelsheikh University, Kafrelsheikh, Egypt.
[k] Pharmacology Unit, Cancer Biology Department, National Cancer Institute (NCI), Cairo University, Cairo, Egypt.

* Corresponding authors:

Wagdy M. Eldehna: Email: wagdy2000@gmail.com
ORCID: 0000-0001-6996-4017

Ahmed A. Al-Karmalawy: Email: akarmalawy@acu.edu.eg
ORCID: 0000-0002-8173-6073

Supplementary Material

Table SI 1: 2 D and 3 D interactions of the examined compounds into the 5Q4 inhibitor binding site of EGFR-Kinase domain.

Comp.	2 D protein interactions	3 D protein interactions
**7**	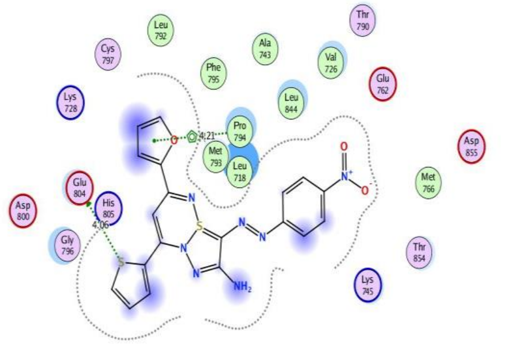	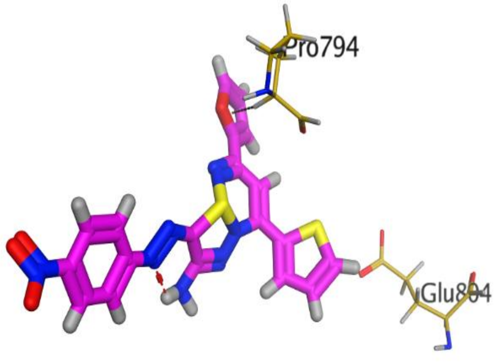
**8**	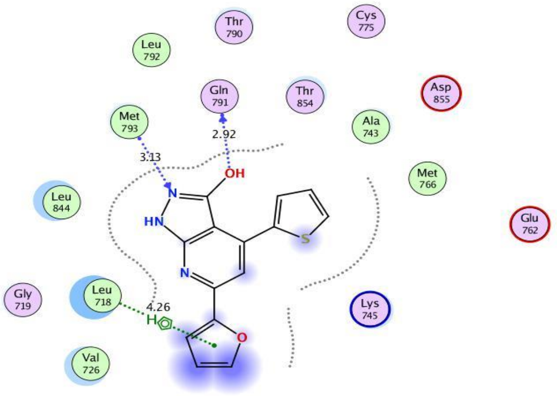	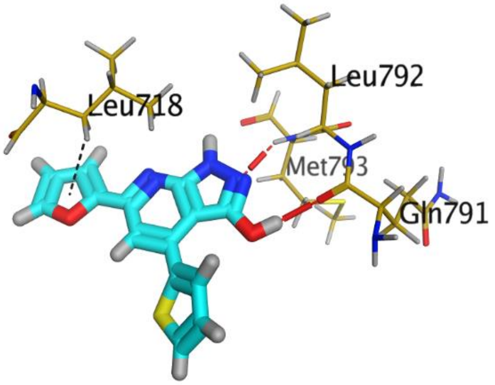
**9**	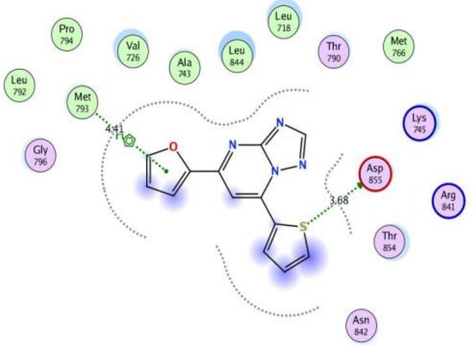	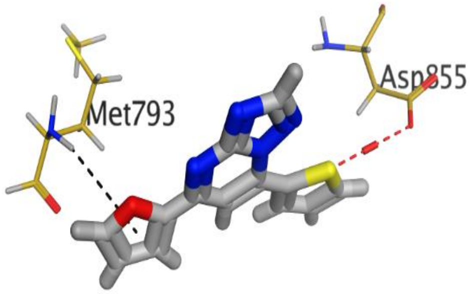

**10**	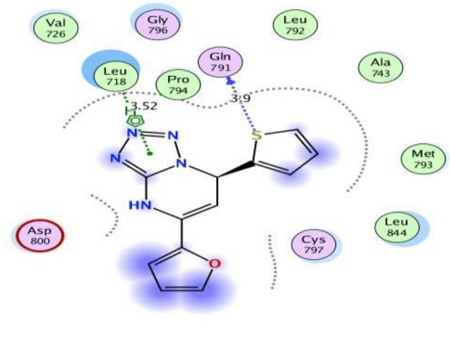 Val 726 Gly 796 Leu 792 Gln 791 Ala 743 Leu 718 Pro 794 3.52 3.9 Met 793 Asp 800 Cys 797 Leu 844	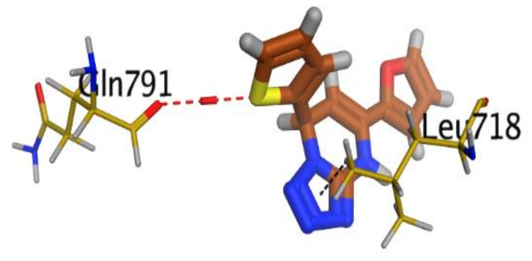 Gln791 Leu718
**11**	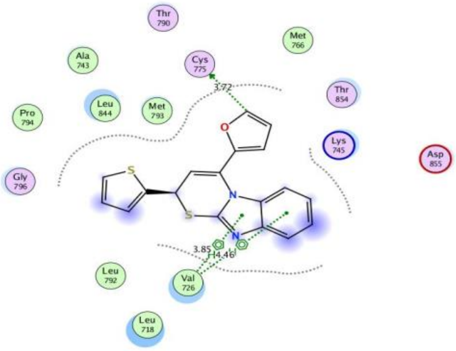 Thr 790 Met 766 Ala 743 Cys 775 3.72 Pro 794 Leu 844 Met 793 Thr 854 Lys 745 Asp 855 Gly 796 3.85 4.46 Leu 792 Val 726 Leu 718	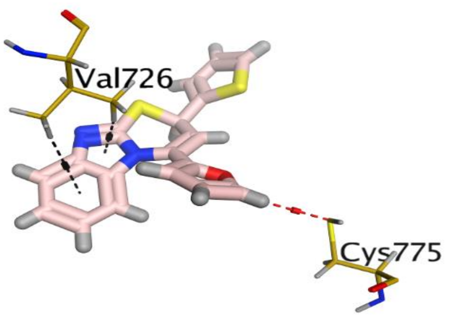 Val726 Cys775
**13**	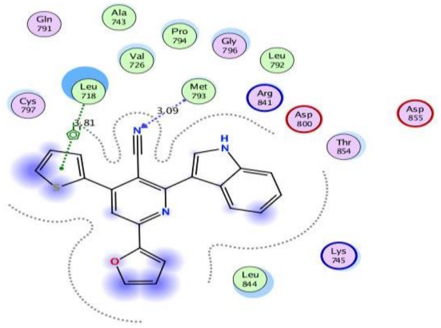 Gln 791 Ala 743 Pro 794 Gly 796 Val 726 Leu 792 Leu 718 Met 793 Arg 841 Cys 797 3.81 3.09 Asp 800 Asp 855 Thr 854 Lys 745 Leu 844	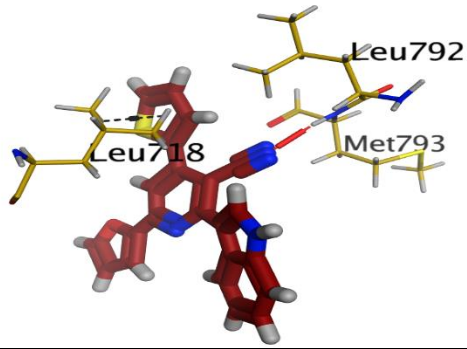 Leu792 Leu718 Met793
**14**	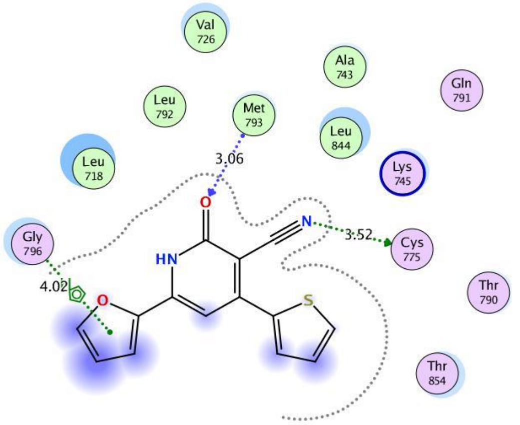 Val 726 Ala 743 Gln 791 Leu 792 Met 793 Leu 844 Leu 718 3.06 Lys 745 Gly 796 3.52 Cys 775 4.02 Thr 790 Thr 854	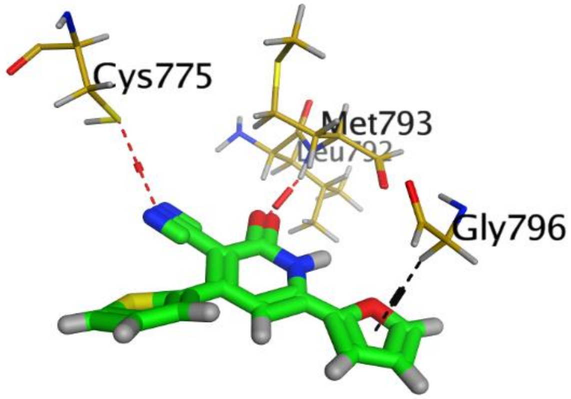 Cys775 Met793 Leu792 Gly796

<table>
<tr><td>15</td><td>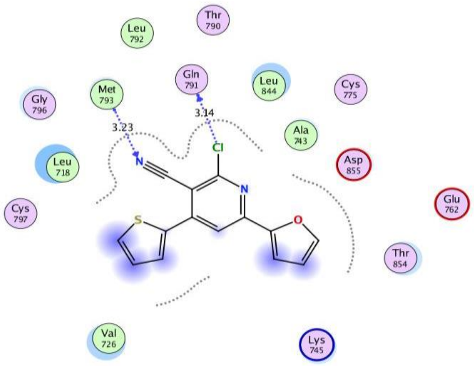</td><td>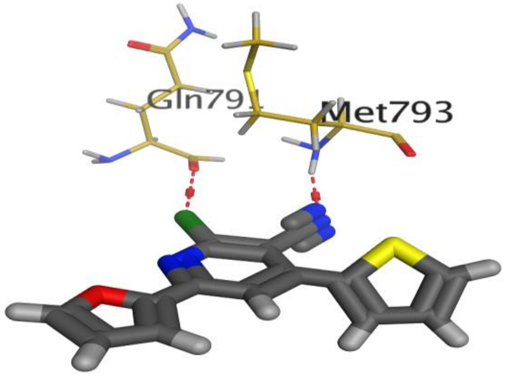</td></tr>
<tr><td>16</td><td>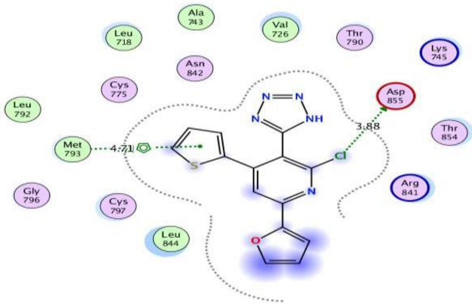</td><td>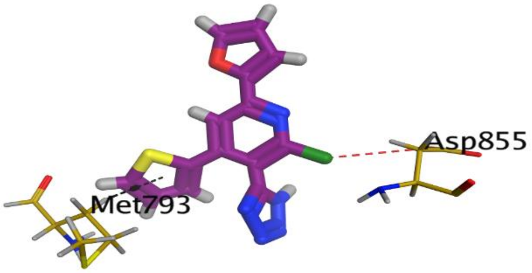</td></tr>
<tr><td>17</td><td>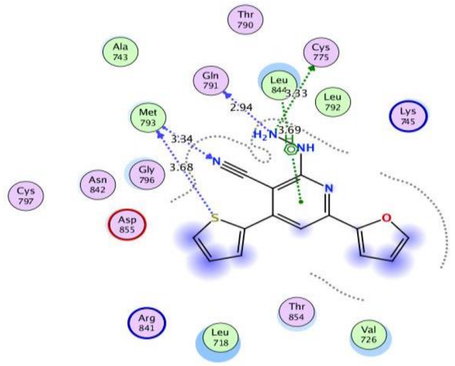</td><td>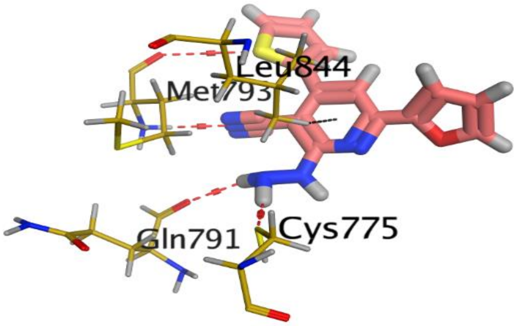</td></tr>
<tr><td>18</td><td>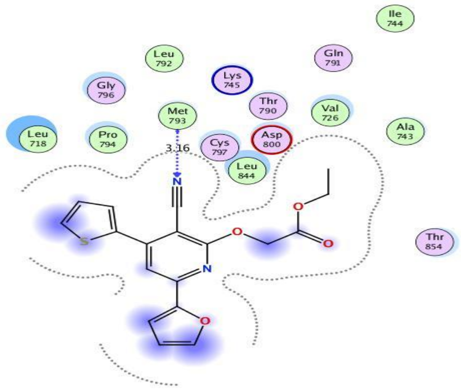</td><td>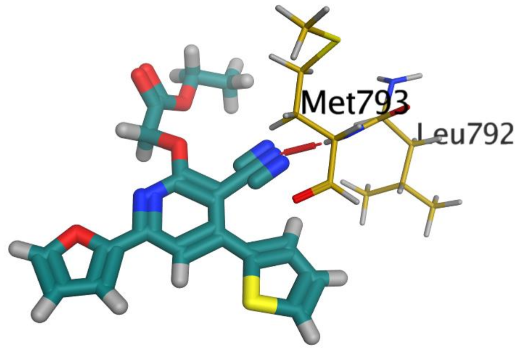</td></tr>
</table>

**19**	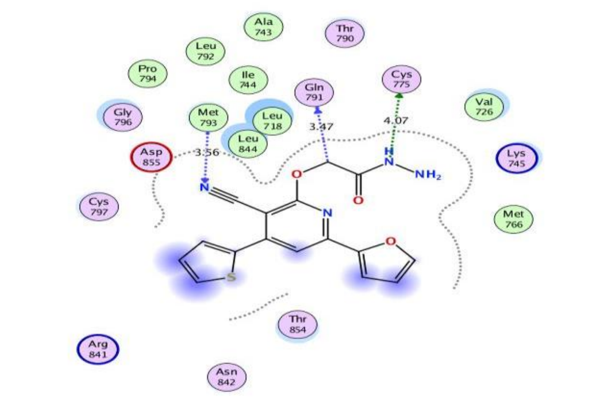	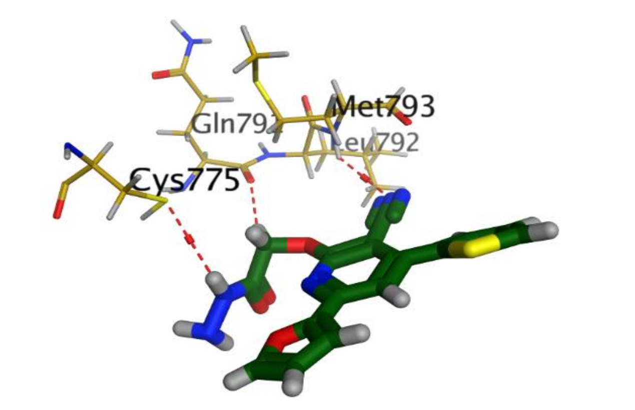
**20**	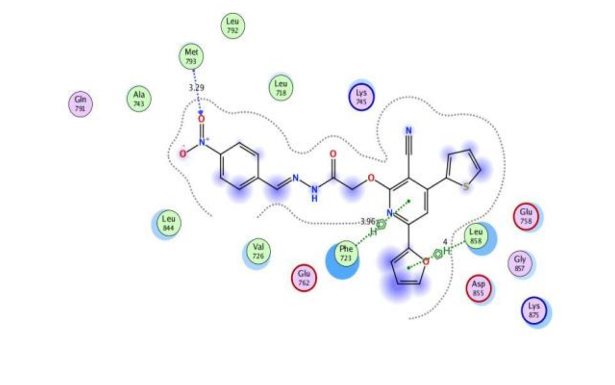	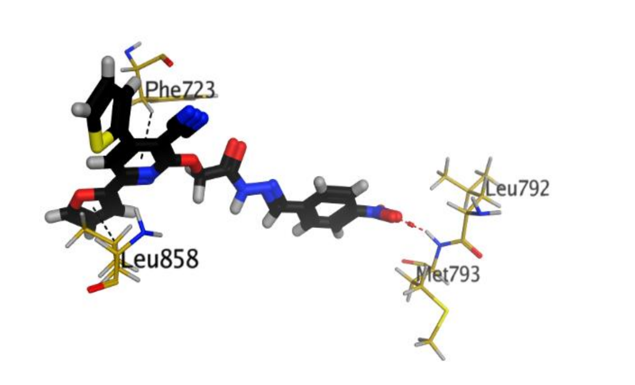
**21**	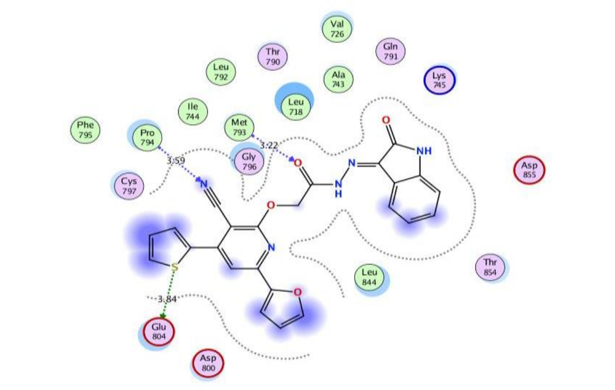	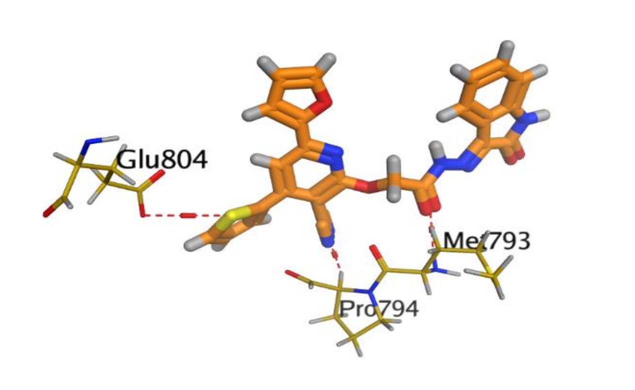
**5Q4**	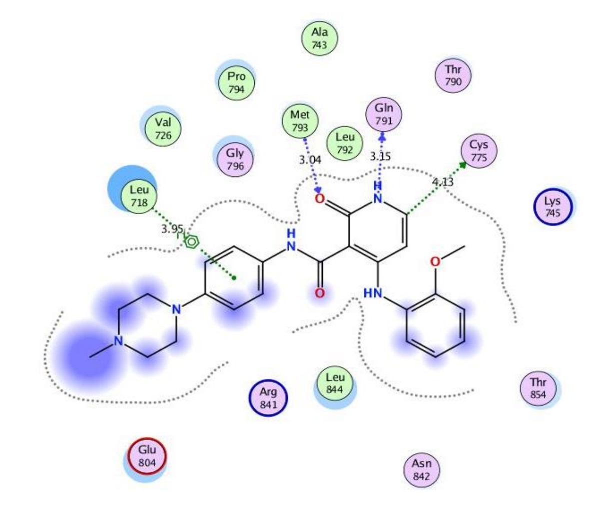	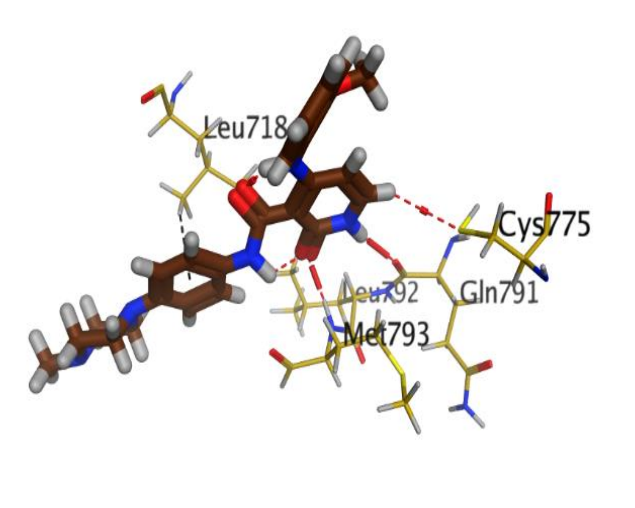

Table SI 2: 3 D protein positioning of the examined compounds into the 5Q4 inhibitor binding site of EGFR-Kinase domain.

Compound	3D protein positioning
**7**	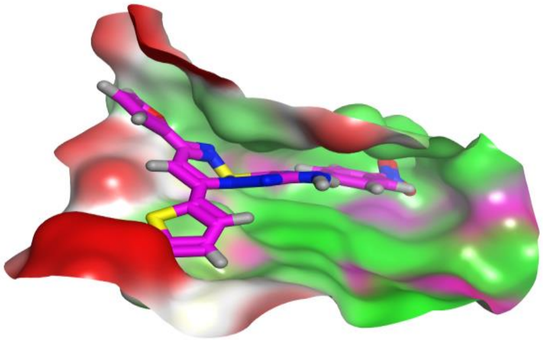
**9**	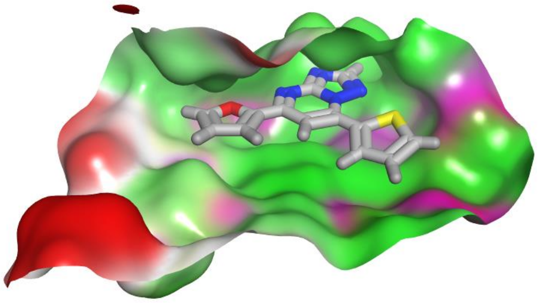
**10**	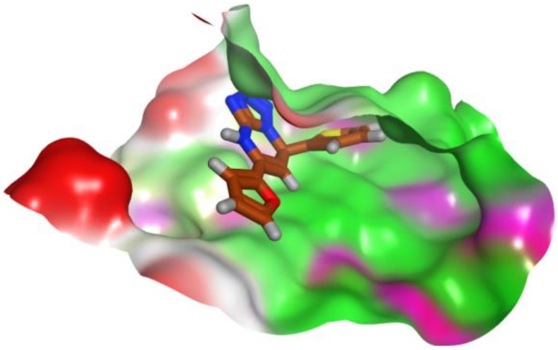
**11**	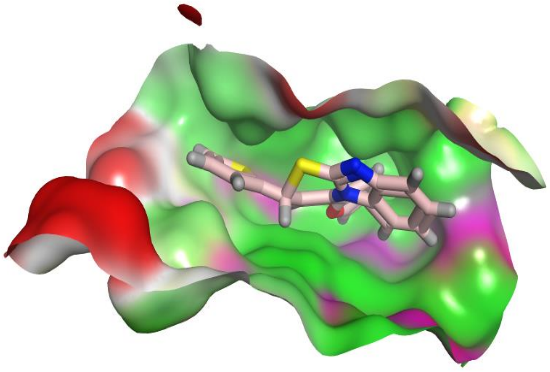

**13**	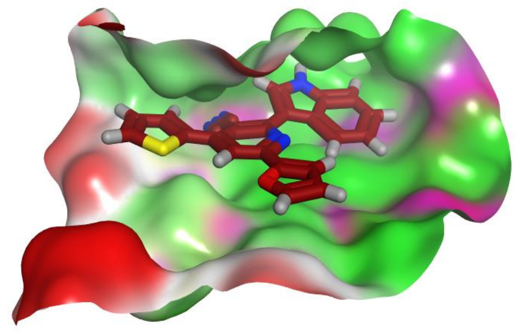
**15**	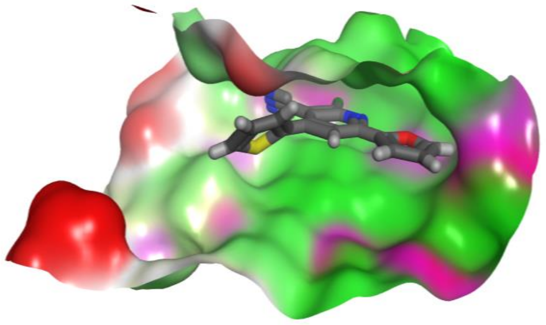
**16**	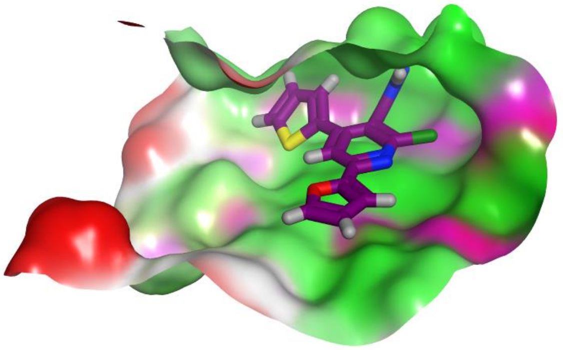
**17**	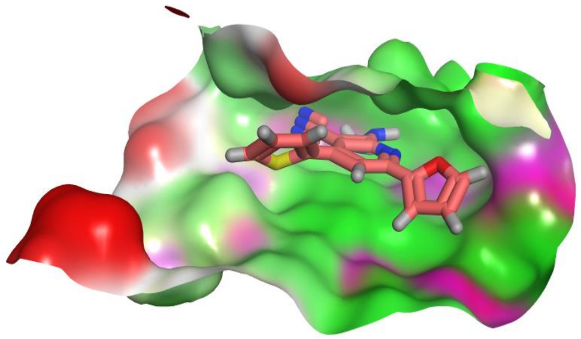

**18**	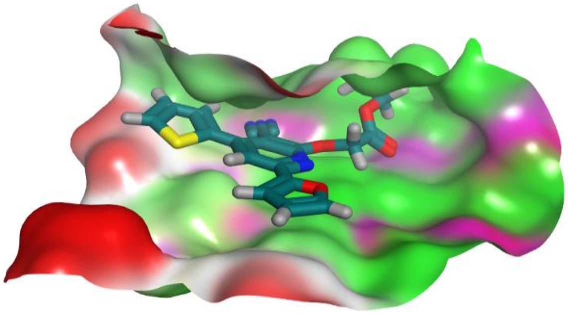
**19**	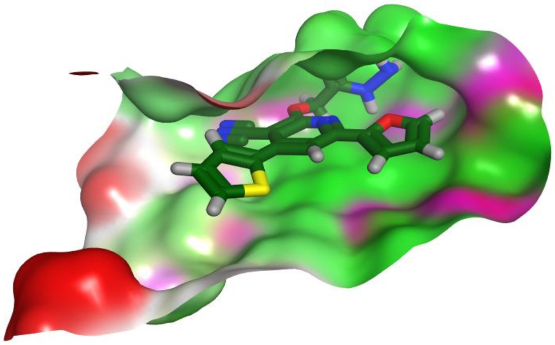
**20**	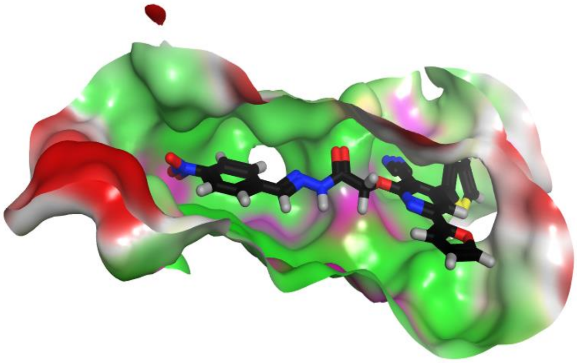
**21**	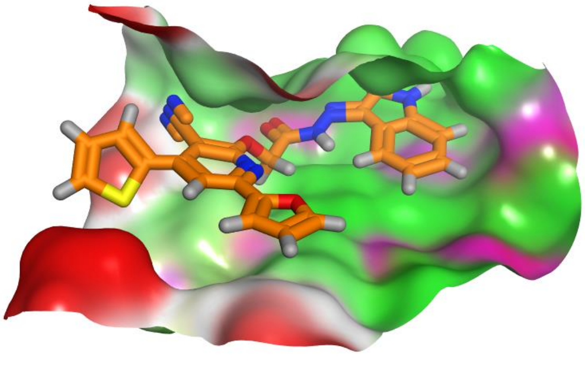

Table SI 3: Receptor binding energies and interactions of the tested compounds into the 5Q4 binding site of EGFR-Kinase.

No.	Compound	Score Kcal/mol	RMSD_Refine	Amino acid bond	Distance A°
**1**	**7**	-6.5487	2.2388	GLU 804 (A) H-donor<br>PRO 794 (A) pi-H	4.06<br>4.21
**2**	**9**	-5.6787	1.4635	ASP 855 (A) H-donor<br>MET 793 (A) pi-H	3.68<br>4.41
**3**	**10**	-5.4536	1.0656	GLN 791 (A) H-donor<br>LEU 718 (A) pi-H	3.90<br>3.52
**4**	**11**	-5.9900	2.2242	CYS 775 (A) H-donor<br>VAL 726 (A) pi-H<br>VAL 726 (A) pi-H	3.72<br>3.85<br>4.46
**5**	**13**	-6.4461	1.4154	MET 793 (A) H-acceptor<br>LEU 718 (A) pi-H	3.09<br>3.81
**6**	**15**	-5.4571	0.9432	GLN 791 (A) H-donor<br>MET 793 (A) H-acceptor	3.14<br>3.23
**7**	**16**	-5.8133	1.4758	ASP 855 (A) H-donor<br>MET 793 (A) pi-H	3.88<br>4.71
**8**	**17**	-5.6533	1.8370	MET 793 (A) H-donor<br>CYS 775 (A) H-donor<br>GLN 791 (A) H-donor<br>MET 793 (A) H-acceptor<br>LEU 844 (A) pi-H	3.68<br>3.33<br>2.94<br>3.34<br>3.69
**9**	**18**	-6.8316	1.8698	MET 793 (A) H-acceptor	3.16
**10**	**19**	-6.3228	1.7075	GLN 791 (A) H-donor<br>CYS 775 (A) H-donor<br>MET 793 (A) H-acceptor	3.47<br>4.07<br>3.56
**11**	**20**	-7.3829	1.4430	MET 793 (A) H-acceptor<br>PHE 723 (A) pi-H<br>LEU 858 (A) pi-H	3.29<br>3.96<br>4.00
**12**	**21**	-7.5845	2.5337	GLU 804 (A) H-donor<br>MET 793 (A) H-acceptor<br>PRO 794 (A) H-acceptor	3.84<br>3.22<br>3.59

Materials and Methods

SI 1: Cytotoxicity screening against human cancer cell lines

In this study, a panel of cell lines was examined for their chemosensitivity. Different concentrations of the synthesized pyridines and pyrimidines derivatives were used in this study (5, 12.5, 25, and 50 µg/ml) for all the examined cell lines. The most sensitive cell line to the treatment regimen was selected and subjected to further investigations to explore the mechanism of this interaction. Human breast tumor cell line (MCF7), human hepatocellular carcinoma cell line (HEPG2), human larynx cell line (HEP2), human lung cancer cell line (H460), human colon cancer cell line (HCT116 and Caco2), human hypopharyngeal cell line (FaDu), and normal cell line Vero were used in this study. It was obtained from the American Type Culture Collection (ATCC, Minnesota, USA). The tumor cell line was maintained at National Cancer Institute (NCI), Cairo, Egypt.

The antitumor activities of pyridines and pyrimidines derivatives and all tested cell lines were evaluated by sulphorhodamine-B (SRB) assay [1]. Briefly, cells were seeded at a density of 3×10^3 cells/well in 96-well microtiter plates. They were left to attach for 24 h before incubation with drugs. Next, cells were treated with different concentrations of 5, 12.5, 25, and 50 µg/ml for MCF7, HEPG2, HEP2, HCT116, Caco2, H460, FaDu, and Vero cells. For each concentration, three wells were used and incubation was continued for 48 h. DMSO was used as a control vehicle (1 % v/v). At the end of incubation, cells were fixed with 20 % trichloroacetic acid and stained with 0.4 % SRB dye. The optical density (O.D.) of each well was measured spectrophotometrically at 570 nm using an ELISA microplate reader (TECAN sunrise™, Germany). The mean survival fraction at each drug concentration was calculated as follows: O.D. of the treated cells/O.D. of the control cells. The IC_{50} (concentration that produces 50% of cell growth inhibition) value of each drug was calculated using sigmoidal dose-response curve-fitting models (Graph Pad Prizm software, version 5) [1].

SI 2: Oxidative stress assessment

SI 2A: Determination of malondialdehyde content (MDA)

Lipid peroxidation products were quantified by measuring MDA level in cell culture lysate of control and treated cells using Lipid Peroxidation (MDA) Assay Kit (Sigma Aldrich Chemical Co., St. Louis, USA) following the manufacturer's instructions. The MDA level was calculated relative to the corresponding protein content. The absorbance was determined at 532 nm using a spectrophotometer (Spectronic, Milton Roy Co., USA).

SI 2B: Determination of superoxide dismutase (SOD)

Lipid peroxidation products were quantified by measuring SOD level in cell culture lysate of control and treated cells by SOD determination kit (Sigma Aldrich Chemical Co., St. Louis, USA) following the manufacturer's instructions. SOD level was calculated relative to the corresponding protein content. The absorbance of the supernatant was determined at 450 nm using a spectrophotometer (Spectronic, Milton Roy Co., USA) [2]. The experiment was carried out three independent times.

SI 2C: Determination of reduced glutathione (GSH) content

Reduced glutathione was determined by adopting Ellman's method. MCF7 and LCC2 cells were harvested, the protein was precipitated with trichloroacetic acid, and Ellman's reagent [5,5-dithiobis-(2-nitrobenzoic acid)] (Sigma Aldrich Chemical Co, St. Louis, USA) was added to the supernatant. The absorbance was read at 405 nm and total SH was calculated as µM of GSH/mg protein [3].

SI 2D: Determination of nitric oxide (NO) content

Nitric oxide was determined in the culture media of the control and treated cells as described before [4]. Briefly, 0.5 ml cold absolute ethanol was added to 250µl culture media then left for 48 h at 4 °C to attain complete protein precipitation followed by centrifugation at 13,000 rpm for 1 h using a cooling centrifuge. 100 µl of nitrate standard solution were serially diluted in duplicate in a 96-well microplate. Then, 100 µl of vanadium chloride were added to each well rapidly followed by 50 µl sulfanilamide and 50µl n-(1-naphthyl) ethylenediamine in 2 N hydrochloric acid. The absorbance at 540 nm was measured spectrophotometrically using an ELISA microplate reader (TECAN SunriseTM, Germany) following an incubation period of 30 min. The level of total nitrite/nitrate was expressed as µM and determined using a standard curve.

SI 3: Cell cycle analysis and apoptosis assay

Annexin V-FITC apoptosis detection kit (Beckman Coulter, California, USA) was used as the manufacturer's recommendation by using (Beckman Coulter EPICS XL flow cytometer, California, USA). On the other hand, the cell cycle DNA index kit (Beckman Coulter, California, USA) was used as the manufacturer's recommendation by using (Beckman Coulter EPICS XL flow cytometer, California, USA). Liver cancer cells (HEPG2) were seeded at a density of 250 X 10^3 cells/mL in RPMI-1640 media. Cell cycle evaluation was performed at 3.8 µg/mL of both compounds 1 and 18. After 48 h, the cells were harvested, stained and DNA content was quantified.

SI 4: EGFR kinase (Wild and T790M) inhibition assay

The most promising cytotoxic compounds (**8** and **14**) were further assessed for their inhibitory potential against both EGFR Wild and EGFRT T790M. The assay protocol was carried out by adding 25 µL of the master mixture: (6 µL Kinase assay buffer + 1 µL ATP (500 µM) + 1 µL PTK substrate + 17 µL water) to each well. Thereafter, 5 µL of the inhibitor solution was added to each well labeled as "test inhibitor". Meanwhile, for the "positive control" and "blank", 5 µL of the same solution without inhibitor (inhibitor buffer) was added. To the wells designated as "blank", add 20 µL of Kinase assay buffer. The amount of EGFR required for the assay was calculated and the enzyme was diluted to 1 ng/µL with Kinase assay buffer. The reaction was initiated by adding 20 µL of diluted EGFR enzyme to the wells designated as "positive control" and "test inhibitor control". The wells were incubated at 30°C for 40 minutes, then 50 µL of Kinase-Glo Max reagent was added to each well. The plate was incubated at room temperature for 15 minutes and luminescence was measured using the microplate reader.

References

1. Skehan, P.; Storeng, R.; Scudiero, D.; Monks, A.; McMahon, J.; Vistica, D.; Warren, J. T.; Bokesch, H.; Kenney, S.; Boyd, M. R., New colorimetric cytotoxicity assay for anticancer-drug screening. *JNCI: Journal of the National Cancer Institute* **1990,** *82* (13), 1107-1112.
2. Aziz, M. A.; Shehab, W. S.; Al-Karmalawy, A. A.; EL-Farargy, A. F.; Abdellattif, M. H., Design, Synthesis, Biological Evaluation, 2D-QSAR Modeling, and Molecular Docking Studies of Novel 1H-3-Indolyl Derivatives as Significant Antioxidants. *International Journal of Molecular Sciences* **2021,** *22* (19), 10396.
3. Ellaman, G., Tissue sulfhydryl group. *Arch Biochem Biophys* **1959,** *82*, 70-7.
4. Miranda, K. M.; Espey, M. G.; Wink, D. A., A rapid, simple spectrophotometric method for simultaneous detection of nitrate and nitrite. *Nitric oxide* **2001,** *5* (1), 62-71.
